# Adjustable positive and negative hygrothermal expansion metamaterial inspired by the Maltese cross

**DOI:** 10.1098/rsos.210593

**Published:** 2021-08-04

**Authors:** Teik-Cheng Lim

**Affiliations:** School of Science and Technology, Singapore University of Social Sciences, Singapore

**Keywords:** auxetic material, Maltese cross, metamaterial, moisture expansion, thermal expansion

## Abstract

A metamaterial that can manifest both positive and negative coefficients of moisture and thermal expansion is presented herein, based on inspiration from the Maltese cross. Each unit of the metamaterial consists of a pair of equal-armed crosses pin-joined at their junctions to permit rotation, but elastically restrained by a bimaterial spiral spring, and four pairs of hinge rods to translate the relative rotational motion of the pair of equal-armed crosses into translational motion of the connecting rods. The effective coefficients of moisture and thermal expansion models were developed for small and large changes in the hygrothermal conditions using infinitesimal (approximate) and finite (exact) motion analyses, respectively, with the former giving constant effective coefficients with respect to environmental changes. Results indicate that the approximate method underestimates the magnitude of both the effective expansion coefficients under cooling and drying but overestimates magnitudes of both coefficients during heating and moistening, and that the change in both expansion coefficients is more drastic during cooling and drying than during heating and moistening. In addition to providing another micro-lattice geometry for effecting expansion coefficients of either signs, this metamaterial exhibits auxetic property.

## 1. Introduction

**Figure 1.**
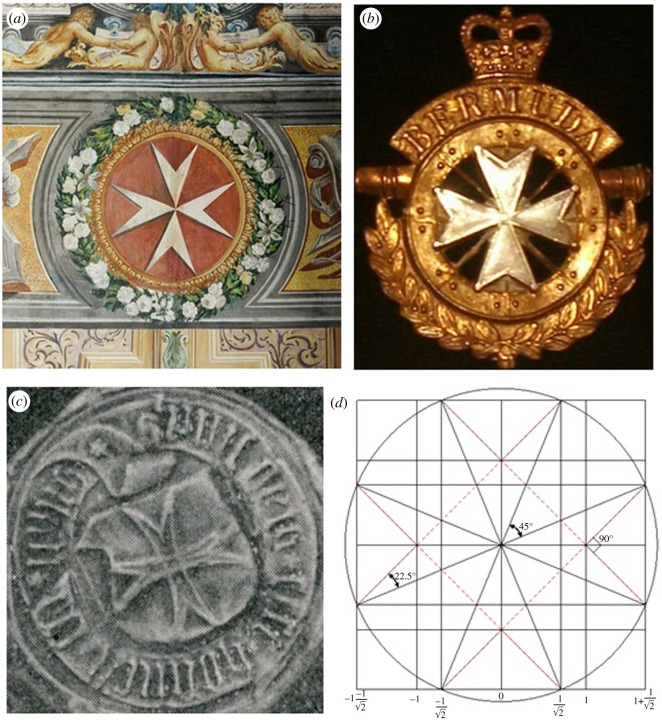
The Maltese cross from the facade of San Giovannino dei Cavalieri, Florence (*a*), badge of the British Army's Bermuda Regiment (*b*), seal of the provost of St John's church, Stockholm, in early-sixteenth century (*c*) and a geometrical description of the Maltese cross (*d*).

There are times when breakthroughs in science are impelled by necessity, at other times by accidental discovery, and still other times advancement is made by drawing inspiration from nature. In recent years, progress in the design of metamaterials has been made by drawing inspiration from Islamic art [1–5]. By contrast, this paper introduces a new mechanical metamaterial by drawing inspiration from the Maltese cross. The Maltese cross has a remarkably rich history (figure 1) and can be found as a relatively common symbol. Variants of the Maltese cross include the Lazarus cross, the Saint Stephen cross and the Huguenot cross, to name a few. The geometrical construction of the Maltese cross, as described by figure 1*d*, forms the basic unit of the currently investigated mechanical metamaterial in its original or reference state.

Metamaterials are materials whose micro-lattices are tailor-made so that effective characteristics are predominantly regulated by their microstructural layout instead of those by the base materials. Metamaterials are artificially made materials that do not exist in nature. The term derives from the Greek word meta, meaning beyond. More specifically, metamaterials are composites that have the desired combination of properties that cannot be obtained by combining the properties of their constituents. The term was coined by Rodger Walser. The reader is referred to books that deal with electromagnetic metamaterials and their applications [6,7], elastic, acoustic and seismic metamaterials [8], and negative mechanical metamaterials [9], as well as special issues pertaining to metamaterials [10,11], to name a few. Gibson [12], Gibson *et al*. [13] and Gibson & Ashby [14] are some of the earliest to publish work on cellular materials with auxetic (negative Poisson's ratio) behaviour. Other pioneering works include those by Almgren [15], Kolpakov [16] and Wojciechowski [17,18], based on mechanical and thermodynamical models. Since 1987, when negative Poisson's ratio foams have been developed by Lakes [19], it is known that materials and structures showing the negative Poisson's ratio do exist in nature. The mechanical response of these materials can be drastically changed depending on the number of applied loads, and auxetic materials are expected to have unusual, possibly enhanced geometrical and mechanical characteristics such as synclastic curvature in bending, deformation-dependent permeability, high shear stiffness, indentation resistance and fracture toughness, and improved damping and sound absorption properties. Strek *et al*. [20] studied the contact problem of a composite plate covered with an auxetic layer. Auxetic material and structures or composite with auxetic phase/layer show better damping properties than materials with positive Poisson's ratio [21]. Some metamaterials have been shown to exhibit temperature dependency of mechanical properties, such as thermoauxeticity [22] or anomalous deformation [23,24]. Owing to the advancement in three-dimensional printing, mechanical metamaterials can be designed in such a manner that the desired overall properties can be achieved. Of interest is a class of metamaterial that manifests counterintuitive behaviour; these materials possess negative values for mechanical properties that are normally positive. Materials typically exhibit a positive coefficient of moisture expansion (CME) as well as a positive coefficient of thermal expansion (CTE), i.e. they dilate upon absorbing moisture and shrink when moisture dissipates from them, as well as expand upon heating and contract when cooled. As such, materials display negative hygrothermal expansion (NHTE) when they demonstrate negative moisture expansion (NME) and/or negative thermal expansion (NTE). Some examples of designed micro-architectures that facilitate NME, NTE and/or negative compressibility (NC) have been made possible by combining materials of non-negative but contrasting expansion coefficients in open-cell micro-truss systems with pin joints or rigid joints [25–39], bimaterial strips [40–46] and counter-rotating parts [47–49]. The category of metamaterials with counter-rotating parts refers to those with scissor-like actions and is a subset of the pin-jointed microstructures. The Hoberman circle consists of rigid pairs of rods that are pin-jointed at their mid-span, and these pairs of rods are arranged in a circle; the metamaterial can be made to exhibit negative thermal expansivity by the inclusion of beam elements of a different material that possesses high CTE [47]. Likewise, the microstructure proposed by Cabras *et al*. [48] possesses Y-shaped elements that have low CTE; a pair of Y-elements are pin-jointed at their junctions to permit relative rotation, which can exhibit structurally overall negative CTE with the incorporation of beam elements of high CTE. Development of metamaterials with such linkage microstructures are now possible with recent advancement in three-dimensional printing technology by incorporating hard and soft materials to mimic pin joints. Although the capability to manifest in a counterintuitive manner—such as negative properties—permits materials and structures to perform in ways that are different from conventional ones, it is of greater practical importance if materials can be tuned to possess both positive and negative properties [49]. In this paper, a tuneable positive hygrothermal expansion (PHTE) and NHTE metamaterial is proposed, in which each unit consists of a pair of equal-armed crosses that are pin-jointed at their centres while their vertices are connected in such a manner that the shape of a Maltese cross is manifested.

## 2. Concept

**Figure 2.**
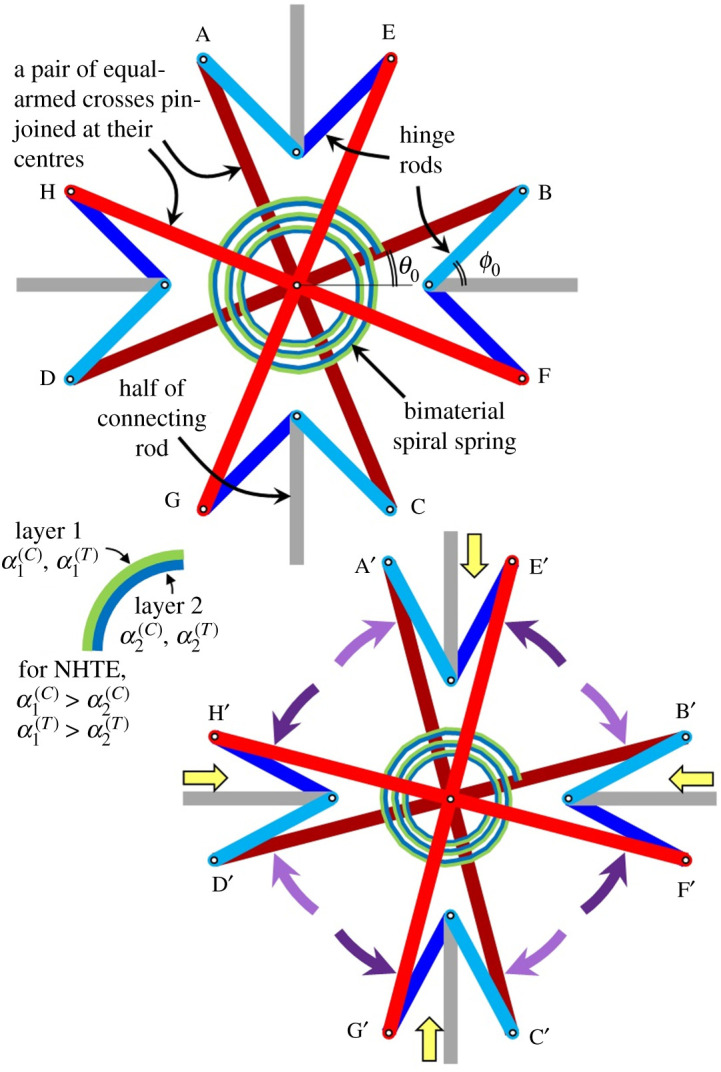
A unit of the Maltese cross metamaterial before (*a*) and after (*b*) temperature and/or moisture increase. NHTE properties are exhibited based on the manner in which the bimaterial spiral spring is attached to the equal-armed crosses.

**Figure 3.**
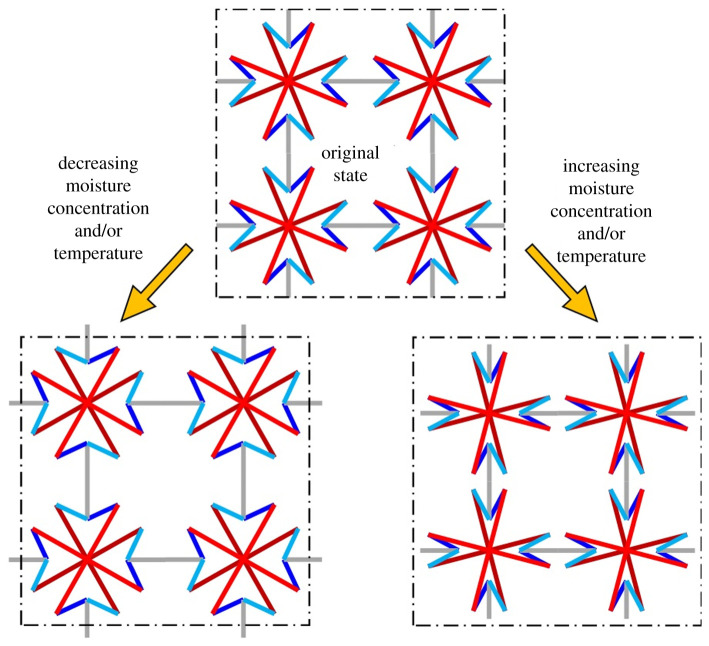
A 2-by-2 array of the Maltese metamaterial in its original state (top) experiencing positive and negative biaxial strains (bottom) arising from decreased and increased curvature of the bimaterial spiral spring. The dashed rectangle indicates the original dimension of the 2-by-2 array. Bimaterial springs and pin joints are not shown for clarity.

The basic unit of the Maltese cross-inspired metamaterial is furnished in figure 2*a*. It is made from two equal-armed crosses that are pin-joined at their junctions to permit free rotation relative to each other. These two equal-armed crosses are linked to eight hinge rods which are attached to four connecting rods so as to establish a connection with four neighbouring units. Let the connecting rods be aligned parallel to the axes such that *θ* is the angle formed between an arm of an equal-armed cross with its nearest axis and *ϕ* is the angle formed between a hinge rod and its nearest axis. At the original state, *θ*_0_ = 22.5°, while the *ϕ*_0_ = 45° so as to reveal the Maltese cross geometry indicated in figure 1*d*. The possibility of NHTE is implemented by the attachment of a spiral spring made from a bimaterial strip with its ends attached to the arms of both equal-armed crosses. The convex and concave layers, hereinafter referred to as layers 1 and 2, respectively, possess contrasting expansion coefficients *α*_1_ > *α*_2_ so that whether the environment's moisture concentration increases Δ*C* > 0 or temperature increases Δ*T* > 0, the bimaterial spring becomes more curved. It is assumed that the crosses, hinge rods and connecting rods are rigid so that they do not elongate, shorten, bend or twist, and that the hinges are frictionless so as to permit free rotation at the pin joints. Based on the manner at which the bimaterial spring is attached as shown in figure 2*a*, the equal-armed cross ABCD rotates clockwise to A′B′C′D′, while the equal-armed cross EFGH rotates anti-clockwise to E′F′G′H′, as indicated in figure 2*b*, such that the arms of the Maltese cross narrow. Accordingly, the hinge rods rotate such that the connecting rods are drawn towards the centre of the Maltese cross, thereby resulting in overall negative in-plane strain. Under an opposing environmental change of drying Δ*C* < 0 and/or cooling Δ*T* < 0, the above-mentioned motions reverse such that the Maltese cross arms broaden and the connecting rods are pushed outward to result in effective positive in-plane strain figure 3. Obviously, the metamaterial exhibits PHTE when *α*_1_ < *α*_2_, thereby availing itself as a material system that can exhibit positive or negative moisture and thermal expansivity.

Since the radius of the spiral spring is defined as the distance from the mid-plane of the bimaterial strip to the centre of the spiral spring, the mid-plane is identified as the interface of the two bimaterial layers if both layers are of equal thickness. The mean radius of the spiral spring is defined as

**Figure 4.**
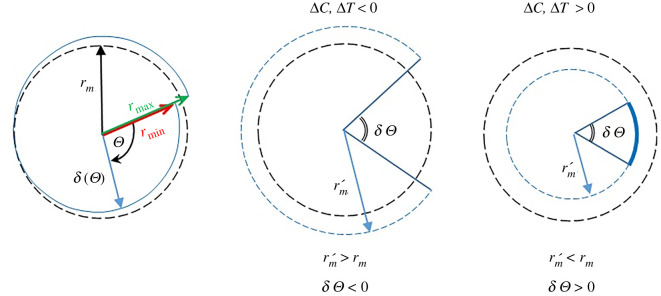
Pictorial description of mean radius *r_m_* of *r* = *r*(*Θ*) for the illustration using of *n* = 1 number of coil, i.e. *Θ* = 2*π* (*a*) for convenience, with increased mean radius of curvature during drying and/or cooling (*b*) and decreased mean radius of curvature during moistening and/or heating (*c*).

2.1 $$r_{m}=\frac{1}{\Theta }\int_{0}^{\Theta }r\;d\Theta ,$$

where *Θ* is the angle swept from the inner end to the outer end of the spiral spring. The use of the mean spiral spring radius is valid when the difference between the maximum and minimum radii is small in comparison to the mean radius, *r*_max_ − *r*_min_ ≪ *r_m_* figure 4*a*. For the sake of clarity, it is instructive to illustrate in figure 4 a spiral spring of *n* = 1 number of coil, i.e. *Θ* = 2*π*. It will later be shown that the value of *n* is not an integer and therefore the use of *n* = 1 is only for the purpose of illustration at this stage. During drying and/or cooling, layer 1 contracts to a greater extent than layer 2 such that the overall curvature of the bimaterial spring decreases, i.e. the mean radius of curvature increases, as shown in figure 4*b*. The produced angular gap of *δΘ* is defined as being negative. During moistening and/or heating, layer 1 elongates to a larger extent than layer 2 so that the overall curvature of the bimaterial spring increases, i.e. the mean radius of curvature decreases, as illustrated in figure 4*c*. The resulting angular overlap of *δΘ* is defined as being positive.

If there are *n* number of coils, the total change in an angle formed by the ends of the spiral spring with its centre is *nδΘ*. By conservation of the arc length of the bimaterial strip $r_{m}(2\pi n)=r_{m}^{\;\prime }(2\pi n+n\delta \Theta )$, we have the altered mean radius of curvature $r_{m}^{\prime }$ as a function of the original mean radius of curvature *r_m_*

2.2 $$r_{m}^{\prime }=\frac{2\pi }{2\pi +\delta \;\Theta }r_{m}.$$

It has previously been shown, in appendix A1 of reference [45], that the change to the arc length is marginal such that the error introduced for assuming constancy in arc length is negligible. For example, in the case of the commonly used copper–steel bimaterial strip, the error for assuming constant arc length is in the order of 10^−3^ [45]. Suppose each equal-armed cross rotates by Δ*θ*, the relative change in angle between a pair of equal-armed crosses is −2Δ*θ*. The negative sign is incorporated based on layer 1 possessing a larger coefficient of expansion than layer 2 and with the outer and inner ends of the spring connected to the equal-armed crosses ABCD and EFGH, respectively, because positive change(s) in moisture concentration Δ*C* and/or temperature Δ*T* decreases the angle *θ*. This arrangement gives rise to NHTE behaviour. The negative sign is to be removed when either layer 1 has the lower expansion coefficient than layer 2 or when the outer and inner ends of the bimaterial spring are connected to the EFGH and ABCD equal-armed crosses, respectively. Consider a bimaterial spiral spring of CME $\alpha_{1}^{\left(C\right)}$ and $\alpha_{2}^{\left(C\right)}$, CTE $\alpha_{1}^{\left(T\right)}$ and $\alpha_{2}^{\left(T\right)}$, Young's modulus *E*_1_ and *E*_2_ and thickness *h*_1_ and *h*_2_ corresponding to layers 1 and 2, respectively, the changes in its mean curvature arising from changes in the environmental moisture concentration Δ*C* and temperature Δ*T* are inferred from Timoshenko's [50] derivation for an initially curved bimetallic strip to give

2.3 $$\frac{1}{r_{m}^{\prime }}-\frac{1}{r_{m}}=\frac{\alpha_{1}^{\left(C\right)}\Delta C_{1}-\alpha_{2}^{\left(C\right)}\Delta C_{2}}{h/2+2/h\;(E_{1}I_{1}+E_{2}I_{2})(\left(1/E_{1}h_{1})+(1/E_{2}h_{2}\right))}$$

and

2.4 $$\frac{1}{r_{m}^{\prime }}-\frac{1}{r_{m}}=\frac{(\alpha_{1}^{\left(T\right)}-\alpha_{2}^{\left(T\right)})\Delta T}{h/2+2/h(E_{1}I_{1}+E_{2}I_{2})(\left(1/E_{1}h_{1})+(1/E_{2}h_{2}\right))},$$

respectively, where $I_{1}=h_{1}^{3}/12$, $I_{2}=h_{2}^{3}/12$ and *h* = *h*_1_ + *h*_2_. It is easy to see that if the bimaterial strip in its reference state is straight (i.e. *r_m_* → ∞), the standard bimaterial strip curving model is recovered upon substitution of $r_{m}^{-1}=0$ into equation (2.4). It must be pointed out that the changes in the temperature of the environment (Δ*T*) as well as in layer 1 (Δ*T*_1_) and in layer 2 (Δ*T*_2_) are common when thermal equilibrium is attained. However, the changes in the moisture concentration of the environment (Δ*C*) as well as in layer 1 (Δ*C*_1_) and in layer 2 (Δ*C*_2_) are in general not common upon hygroscopic equilibrium due to the different extent of moisture absorptivity for different material. For this reason, equation (2.3) differs slightly from equation (2.4). In addition, the CME is dimensionless unlike CTE due to their definitions. The moisture concentration in a solid is defined as

2.5 $$C=\frac{m}{M}\times 100,$$

where *m* is the mass of water in a solid of dry mass *M*, thus *C* is dimensionless due to the ratio *m*/*M*. For consistency, the moisture concentration in the environment is similarly defined herein as expressed by equation (2.5), but with *m* being the mass of environmental moisture enclosed within a volume of air of dry mass *M*. As such, the changes in moisture concentration for both the environment and solid

2.6 $$\Delta C=\frac{\Delta m}{M}\times 100,$$

are similarly dimensionless. Since the moisture strain is defined as $\varepsilon^{\left(C\right)}=\alpha^{\left(C\right)}\Delta C$, it follows that the CME *α*^(*C*)^ is dimensionless. Notwithstanding its dimensionless coefficient, the subsequent analysis is developed for the effective CME due to the more generic form of equation (2.3) in comparison to equation (2.4), as it will later be shown that the effective CME can be easily reduced to the effective CTE. Substituting equation (2.2) into the LHS of equation (2.3) and considering equal layer thickness in the bimaterial spring *h*_1_ = *h*_2_ = *h*/2, we have the description of angular change

2.7 $$\Delta \theta =\pm \frac{24n\pi r_{m}}{h}\frac{\alpha_{1}^{\left(C\right)}\Delta C_{1}-\alpha_{2}^{\left(C\right)}\Delta C_{2}}{14+(E_{1}/E_{2})+(E_{2}/E_{1})},$$

where the upper and lower signs indicate positive and NHTE properties depending on the expansion coefficient of layers 1 and 2, as well as the manner of spiral spring attachment on the equal-armed crosses. For obvious reasons, the section on small deformation analysis adopts *dθ* as well as $dC_{1}\;\&\;dC_{2}$, but reverts to Δ*θ* and $\Delta C_{1}\;\&\;\Delta C_{2}$ in the section on large deformation analysis.

## 3. Small hygrothermal change

**Figure 5.**
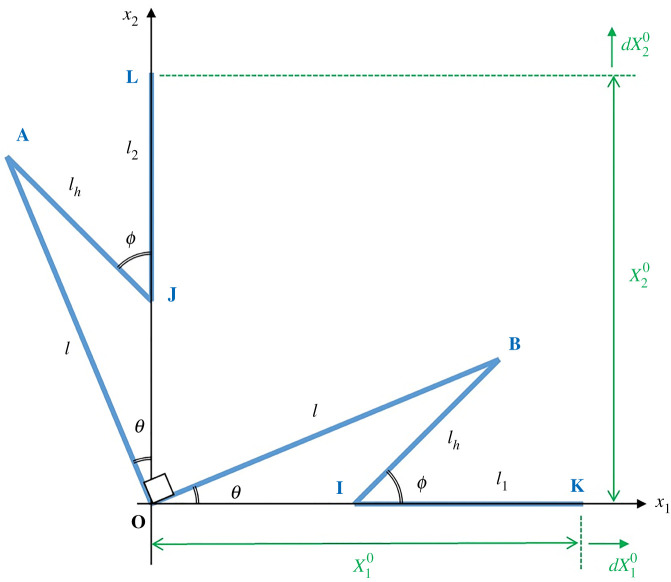
Diagram for small motion analysis of the Maltese cross metamaterial.

Figure 5 depicts a quarter of the metamaterial unit for establishing the effective CME based on the assumption of small change in moisture concentration, whereby AOB, as part of an equal-armed cross, rotates as a rigid body with the change in curvature of the bimaterial spiral spring. By virtue of symmetry due to an opposing rotational motion from the other equal-armed cross, the connecting rods IK and JL are confined to translational motion along the *Ox*_1_ and *Ox*_2_ axes, respectively. The accompanying rotation of the hinge rods can, therefore, be expressed in terms of the equal-armed cross rotation. Let *l* be the length for one arm of the equal-armed cross, *l_h_* the length of the hinge rods, while *l*_1_ and *l*_2_ are the half-lengths of the connecting rods oriented parallel to the *Ox*_1_ and *Ox*_2_ axes, respectively. The relationship between *l_h_* and *l* can be obtained by projecting their lengths on the corresponding axes, which in turn leads to the relationship between the rotational angles of the hinge rods *dϕ* and the equal-armed crosses *dθ*

3.1 $$\begin{array}{rl} l_{h}\sin\phi = & l\sin\theta \\ l_{h}\cos\phi \;d\phi = & l\cos\theta \;d\theta \end{array}\},$$

whereby

3.2 $$0<\theta <\phi <\frac{\pi }{2}.$$

Substituting *ϕ*_0_ = 45° and *θ*_0_ = 22.5° for the original state gives

3.3 $$\begin{array}{rl} l_{h}= & \sqrt{\frac{2-\sqrt{2}}{2}}\;l\\ d\phi = & \sqrt{\frac{2+\sqrt{2}}{2-\sqrt{2}}}\;d\theta \end{array}\}.$$

The second of equation (3.3) can be expressed as *dϕ*/*dθ* = *δ_S_* where *δ_S_* is the silver ratio defined as $\sqrt{2}+1$.

With reference to figure 5, the distance OK and OL as well as their incremental displacements along the corresponding axes can be collectively described as

3.4 $$\begin{array}{rl} X_{i}= & l\cos\theta -l_{h}\cos\phi +l_{i}\\ dX_{i}= & -l\sin\theta \;d\theta +l_{h}\sin\;\phi d\phi \end{array}\}.$$

Substituting *ϕ*_0_ = 45° and *θ*_0_ = 22.5° for the original state and using equation (3.3), we have

3.5 $$\begin{array}{cc} X_{i}^{0}=\; & \frac{\sqrt{2+\sqrt{2}}-\sqrt{2-\sqrt{2}}}{2}l+l_{i}\;\\ dX_{i}^{0}=\; & \frac{\sqrt{2+\sqrt{2}}-\sqrt{2-\sqrt{2}}}{2}l\;d\theta \; \end{array}\},$$

from which the strain is obtained as

3.6 $$\varepsilon_{ii}^{0}=\frac{dX_{i}^{0}}{X_{i}^{0}}=\frac{\left(\sqrt{2+\sqrt{2}}-\sqrt{2-\sqrt{2}}\right)/2\;d\theta }{\left(\sqrt{2+\sqrt{2}}-\sqrt{2-\sqrt{2}}\right)/2+(l_{i}/l)}.$$

From the definition of small effective moisture strain along an axis $\varepsilon_{ii}^{\left(C\right)}=\alpha_{ii}^{\left(C\right)}dC$ due to a small change in environmental moisture concentration, one obtains

3.7 $$\alpha_{ii}^{\left(C\right)}=\frac{\left(\sqrt{2+\sqrt{2}}-\sqrt{2-\sqrt{2}}\right)/2\;}{\left(\sqrt{2+\sqrt{2}}-\sqrt{2-\sqrt{2}}\right)/2+(l_{i}/l)}\frac{d\theta }{dC}\;.$$

Substituting equation (2.7) into equation (3.7) gives the on-axis effective CME

3.8 $$\alpha_{ii}^{\left(C\right)}=\pm \frac{24n\pi r_{m}}{h}\frac{\left(\sqrt{2+\sqrt{2}}-\sqrt{2-\sqrt{2}}\right)/2\;}{\left(\sqrt{2+\sqrt{2}}-\sqrt{2-\sqrt{2}}\right)/2+(l_{i}/l)}\frac{\alpha_{1}^{\left(C\right)}(dC_{1}/dC)-\alpha_{2}^{\left(C\right)}(dC_{2}/dC)\;}{14+(E_{1}/E_{2})+(E_{2}/E_{1})}.$$

The on-axis effective CTE can be obtained by making the following substitutions

3.9 $$\left[\begin{array}{c} \begin{array}{c} \alpha_{1}^{\left(C\right)}\\ \alpha_{2}^{\left(C\right)} \end{array}\\ \begin{array}{c} dC_{1}\\ dC_{2}\\ dC \end{array} \end{array}\right]\to \left[\begin{array}{c} \begin{array}{c} \alpha_{1}^{\left(T\right)}\\ \alpha_{2}^{\left(T\right)} \end{array}\\ \begin{array}{c} dT\\ dT\\ dT \end{array} \end{array}\right],$$

on equation (3.8) to give rise to

3.10 $$\alpha_{ii}^{\left(T\right)}=\pm \frac{24n\pi r_{m}}{h}\frac{\left(\sqrt{2+\sqrt{2}}-\sqrt{2-\sqrt{2}}\right)/2\;}{\left(\sqrt{2+\sqrt{2}}-\sqrt{2-\sqrt{2}}\right)/2+(l_{i}/l)}\frac{\alpha_{1}^{\left(T\right)}-\alpha_{2}^{\left(T\right)}\;}{14+(E_{1}/E_{2})+(E_{2}/E_{1})}.$$

It can be seen that the on-axis effective CME is influenced by the change in moisture concentrations in the environment (*dC*), in layer 1 (*dC*_1_) and in layer 2 (*dC*_2_), but the on-axis effective CTE is independent from the change in temperature *dT*, thereby availing the latter as a material property that is independent from the environmental condition for infinitesimal change in temperature.

## 4. Large hygrothermal change

**Figure 6.**
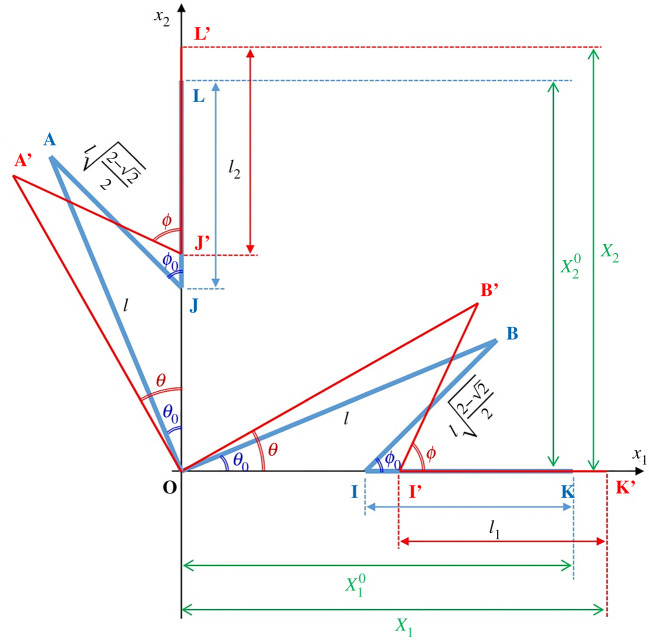
Diagram for large motion analysis of the Maltese cross metamaterial.

Figure 6 shows two sets of a quarter unit of the metamaterial for large deformation analysis, whereby a change in the bimaterial spiral spring's curvature rotates AOB to A′OB′ by an angle Δ*θ* = *θ* − *θ*_0_, during which IK and JL slide along the corresponding axes to I′K′ and J′L′, respectively. The simultaneous motion of hinge rods IB and JA to I′B′ and J′A′, respectively, changes their subtending angles from *ϕ*_0_ to *ϕ* = *ϕ*_0_ + Δ*ϕ*. The total strain *ε_ii_* along an axis is related to the incremental strain along the same axis *dε_ii_* = *dX_i_*/*X_i_* as $\varepsilon_{ii}=\int d\varepsilon_{ii}$. Hence substituting from the firsts of equations (3.4) and (3.5) and using the first of equation (3.3) leads to

4.1 $$\varepsilon_{ii}=\int_{X_{i}^{0}}^{X_{i}}\frac{dX_{i}}{X_{i}}=\ln\frac{\cos\theta -\sqrt{(2-\sqrt{2})/2}\cos\phi +l_{i}/l}{\left(\sqrt{2+\sqrt{2}}-\sqrt{2-\sqrt{2}})/2+(l_{i}/l\right)}.$$

The angle *ϕ* can be obtained by eliminating *l* and *l_h_* from the firsts of equations (3.1) and (3.3) to yield

4.2 $$\phi =\sin^{-1}\left(\sqrt{\frac{2}{2-\sqrt{2}}}\sin\theta \right).$$

The maximum value for the angle *θ* can be obtained by substituting *ϕ* = 90° so that *θ*_max_ = 0.57186 radians or 32.765°.

Substituting equation (4.2) into equation (4.1) and considering the definition of on-axis moisture strain $\varepsilon_{ii}^{\left(C\right)}=\alpha_{ii}^{\left(C\right)}\Delta C$ arising from a large change in environmental moisture concentration Δ*C*, one obtains the effective on-axis CME

4.3 $$\alpha_{ii}^{\left(C\right)}=\frac{1}{\Delta C}\ln\frac{\cos\theta -\sqrt{(2-\sqrt{2})/2}\cos\left\{\sin^{-1}\left(\sqrt{2/(2-\sqrt{2})}\sin\theta \right)\right\}+(l_{i}/l)}{\left(\sqrt{2+\sqrt{2}}-\sqrt{2-\sqrt{2}})/2+(l_{i}/l\right)},$$

where

4.4 $$\theta =\theta_{0}+\Delta \theta =\frac{\pi }{8}\pm \frac{24n\pi r_{m}}{h}\frac{\alpha_{1}^{\left(C\right)}\Delta C_{1}-\alpha_{2}^{\left(C\right)}\Delta C_{2}}{14+(E_{1}/E_{2})+(E_{2}/E_{1})}.$$

The effective on-axis CTE for large temperature change can be obtained from equations (4.3) and (4.4) by adopting the substitutions described by equation (3.9) to yield

4.5 $$\alpha_{ii}^{\left(T\right)}=\frac{1}{\Delta T}\ln\frac{\cos\theta -\sqrt{(2-\sqrt{2})/2}\cos\left\{\sin^{-1}\left(\sqrt{2/(2-\sqrt{2})}\sin\theta \right)\right\}+l_{i}/l}{\left(\sqrt{2+\sqrt{2}}-\sqrt{2-\sqrt{2}})/2+(l_{i}/l\right)},$$

where

4.6 $$\theta =\theta_{0}+\Delta \theta =\frac{\pi }{8}\pm \frac{24n\pi r_{m}}{h}\frac{(\alpha_{1}^{\left(T\right)}-\alpha_{2}^{\left(T\right)})\Delta T}{14+(E_{1}/E_{2})+(E_{2}/E_{1})}.$$

Unlike the case of small deformation, the CTE for large change in temperature is influenced by Δ*T*. It can be seen that equation (4.3) for $\alpha_{ii}^{\left(C\right)}$ and equation (4.5) for $\alpha_{ii}^{\left(T\right)}$ are undefined when Δ*C* = 0 and Δ*T* = 0, respectively. For this reason, the plotting of effective CME and CTE under a wide range of environmental change are defined by equations (4.3) and (4.5) for Δ*C* ≠ 0 and Δ*T* ≠ 0, but rely on equations (3.8) and (3.10) for Δ*C* = 0 and *dT* = 0.

To provide a generic appreciation on how the small deformation analysis deviates from the large deformation analysis in terms of the angular change of *ϕ* vis-à-vis that of *θ*, equation (4.2) can be expressed in the form

4.7 $$\Delta \phi =-\frac{\pi }{4}+\sin^{-1}\left(\sqrt{\frac{2}{2-\sqrt{2}}}\sin\theta \right),$$

for comparing against the second of equation (3.3). Alternatively, the second of equation (3.3) for small deformation can be written as

**Figure 7.**
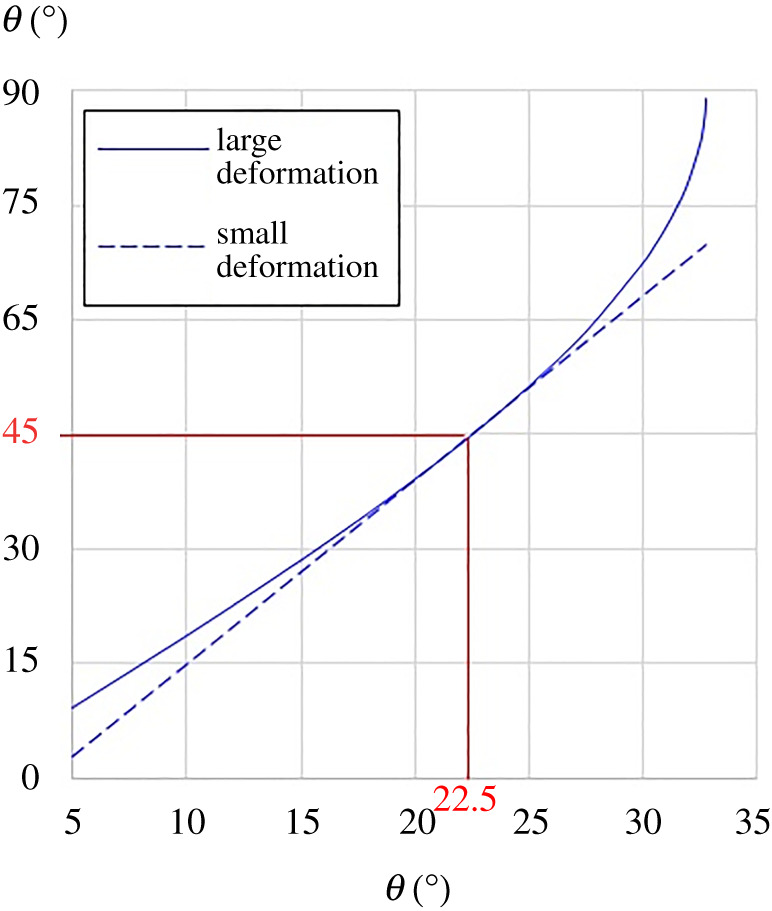
Deviation of small deformation analysis from the large deformation analysis based solely on the angular changes.

4.8 $$\phi =\frac{\pi }{4}+\sqrt{\frac{2+\sqrt{2}}{2-\sqrt{2}}}\;d\theta ,$$

for comparing against equation (4.2). A visual depiction on how the small deformation analysis deviates from the large deformation analysis based only on the angular changes is shown in figure 7 using equations (4.2) and (4.8). Plots of effective CMEs and CTEs, including the effects of small deformation assumption (approximate) vis-à-vis large deformation consideration (exact) including the effects from Δ*C* and Δ*T*, are discussed in the next section.

## 5. Results and discussion

For the sake of brevity, the subsequent results were calculated based on $\alpha_{1}^{\left(C\right)}>\alpha_{2}^{\left(C\right)}$ and $\alpha_{1}^{\left(T\right)}>\alpha_{2}^{\left(T\right)}$, with the spiral springs attached in the manner furnished in figure 2 so as to give NHTE properties, i.e. the lower signs of equations (2.7), (3.8), (3.10), (4.4) and (4.6) are employed. Perusal to the analyses indicates that the effective on-axes CME and CTE models are proportional to the number of coils *n* and the mean radius *r_m_* but inversely proportional to the thickness *h* of the bimaterial spiral springs. Hence, investigations are made on the effect of the difference in the bimaterial layers' expansion coefficients $\left(\alpha_{1}^{\left(T\right)}-\alpha_{2}^{\left(T\right)}\right)$ or $\left(\alpha_{1}^{\left(C\right)}-\alpha_{2}^{\left(C\right)}\right)$, array spacing *l*_1_/*l* or *l*_2_/*l*, the off-axis direction at an angle *ζ* counter-clockwise from the *Ox*_1_ axis, the effect from temperature change sign and magnitude Δ*T* and the effect of different moisture absorptivity Δ*C*_1_/Δ*C* or Δ*C*_2_/Δ*C*. Since both ends of the spiral spring are attached to the pair of equal-armed crosses, which are offset by an angle of 45°, the coil number is

5.1 $$n=\frac{2N-1}{8},$$

where *N* = 1, 2, 3, … is a positive integer. In practice, the number of coil must be *n* > 1. For illustration purposes, the subsequent results are based on *n* = 2.875 as indicated in figure 2. The mean radius of the bimaterial spiral spring would practically be two or three orders greater than its thickness, i.e. 0.001*r_m_* < *h* < 0.01*r_m_*. Hence, a choice of *h* = 0.0069*r_m_* would give 24*nπr_m_*/*h* = 10 000*π* for the convenience of calculating equations (3.8), (3.10), (4.4) and (4.6). The most common bimaterial strip for responding to temperature change is made from copper–steel (C–S) pair [51–54]. To evaluate the effect of CTE difference, other material pairs such as tungsten–silicon carbide (T–SC) pair and brass–titanium (B–T) pair are included [3,45]. The relevant properties for these materials are furnished in table 1, in which layers 1 and 2 denote the material of higher and lower CTEs, respectively.

**Table 1.** Material properties for the tungsten–silicon carbide (T–SC), copper–steel (C–S) and brass–titanium (B–T) bimaterial strips.

bimaterial pair	CTE	Young's modulus	Poisson's ratio
tungsten	$\alpha_{1}^{\left(T\right)}=4.5\times 10^{-6}\;K^{-1}$	*E*_1_ = 405 GPa	*v*_1_ = 0.280
silicon carbide	$\alpha_{2}^{\left(T\right)}=2.77\times 10^{-6}\;K^{-1}$	*E*_2_ = 450 GPa	*v*_2_ = 0.360
copper	$\alpha_{1}^{\left(T\right)}=17\times 10^{-6}\;K^{-1}$	*E*_1_ = 117 GPa	*v*_1_ = 0.360
steel	$\alpha_{2}^{\left(T\right)}=12\times 10^{-6}\;K^{-1}$	*E*_2_ = 200 GPa	*v*_2_ = 0.300
brass	$\alpha_{1}^{\left(T\right)}=19\times 10^{-6}\;K^{-1}$	*E*_1_ = 112.5 GPa	*v*_1_ = 0.357
titanium	$\alpha_{2}^{\left(T\right)}=8.6\times 10^{-6}\;K^{-1}$	*E*_2_ = 110.3 GPa	*v*_2_ = 0.300

**Figure 8.**
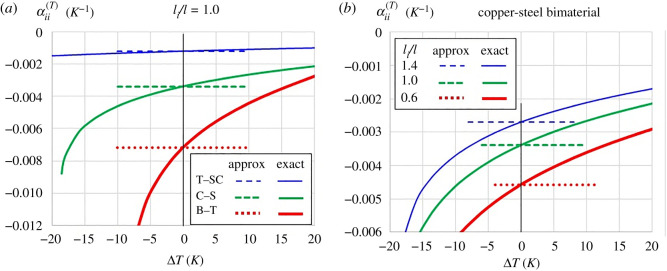
Effect of bimaterial layers' CTE difference (*a*) and spacing (*b*) on the on-axis effective CTE using small deformation model (approx) and large deformation model (exact).

The outcome from the use of different pairs of bimaterial strips as the spiral spring on the effective on-axis CTE is shown in figure 8*a* for the case where *l_i_*/*l* = 1, wherein the $\alpha_{ii}^{\left(T\right)}$ is independent from Δ*T* while the large deformation model reveals a more negative effective on-axis CTE during cooling but less negative during heating. This suggests that the small deformation model underestimates and overestimates the negativity of $\alpha_{ii}^{\left(T\right)}$ during cooling and heating, respectively. A similar response is observed when the effective on-axis CTE is plotted for variation in spacing between a Maltese cross unit from its immediate neighbour along the direction of interest, as shown in figure 8*b*. As expected, the calculated $\alpha_{ii}^{\left(T\right)}$ by both small and large deformation models show that the effective on-axis CTE is more negative when the Maltese cross units are packed closer together. The results also indicate that the small deformation model is accurate only for infinitesimal change in temperature, and that the small deformation model underestimates and overestimates $\alpha_{ii}^{\left(T\right)}$ magnitude under decreasing and increasing temperature, respectively.

Due to the prevalence of the C-S bimaterial in practice, subsequent results for the effective CTEs are based on this bimaterial. The off-axis CTE can be plotted using transformation of axis for strain

**Figure 9.**
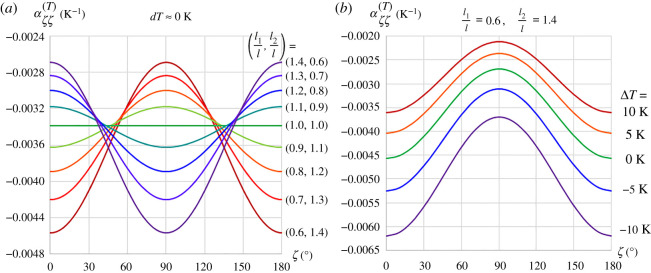
Influence of array aspect ratio for infinitesimal change in temperature (*a*) and various temperature change at a fixed array aspect ratio (*b*) on the effective off-axis CTE.

5.2 $$\varepsilon_{\zeta \zeta }=\varepsilon_{11}\cos^{2}\zeta +\varepsilon_{22}\sin^{2}\zeta +2\varepsilon_{12}\sin\zeta \cos\zeta ,$$

where *ζ* is the direction of consideration measured anti-clockwise from the *Ox*_1_ axis, while *ε*_12_ = 0 due to symmetry of deformation about both axes. Substitution of equation (3.10) where *i* = 1, 2 for the direction along the *Ox*_1_ and *Ox*_2_ axes and $\alpha_{ii}^{\left(T\right)}=\varepsilon_{ii}^{\left(T\right)}/dT$ for small change in temperature, a family of off-axis CTE is plotted in figure 9*a* for various rectangular array aspect ratios. It is noteworthy that while the value of *l*_1_/*l* + *l*_2_/*l* is conserved the deviation from square array causes the mean $\alpha_{\zeta \zeta }^{\left(T\right)}$ value to be more negative. To observe the effect of temperature change magnitude and its sign, the off-axis CTE was plotted with *l*_1_/*l* = 0.6 and *l*_2_/*l* = 1.4 using equations (4.5) and (4.6), as shown in figure 9*b* for Δ*T* = 10*K*, 5*K*, − 5*K*, −10*K*, and equation (3.10) for Δ*T* = 0*K*. Plotted results reveal that the effective CTE becomes less negative and more negative under heating and cooling, respectively, wherein the magnitude of shift in $\alpha_{\zeta \zeta }^{\left(T\right)}$ is greater under cooling than under heating for equal |Δ*T*|. In addition, the undulation of the effective off-axis CTE with reference to angle *ζ* is more pronounced under cooling than under heating.

The CMEs of metals are zero, and so the effective CME of the metamaterial can be put into effect by using polymeric bimaterials. The CME of polymers can range from 2 × 10^−3^ to 5 × 10^−3^ [55]. Suppose both layers of the bimaterial spiral spring are made from the same material $E_{1}/E_{2}=\alpha_{1}^{\left(C\right)}/\alpha_{2}^{\left(C\right)}=1$ but with layer 2 being covered by water-proof coating, i.e. Δ*C*_2_ = 0. This abridges equation (3.8) to

5.3 $$\alpha_{ii}^{\left(C\right)}=\pm \frac{3n\pi r_{m}}{2h}\frac{\sqrt{2+\sqrt{2}}-\sqrt{2-\sqrt{2}}\;}{\sqrt{2+\sqrt{2}}-\sqrt{2-\sqrt{2}}+2(l_{i}/l)}\alpha_{1}^{\left(C\right)}\frac{dC_{1}}{dC},$$

for small deformation model, while equations (4.3) and (4.4) for large deformation model can be combined to give

**Figure 10.**
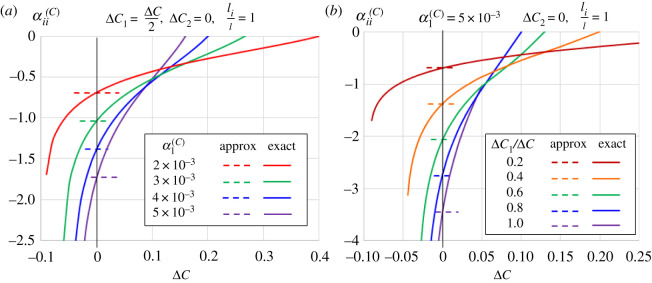
Effect of bimaterial's CME difference (*a*) and absorptivity (*b*) on the effective on-axis CME where layer 2 is water-proofed.

5.4 $$\alpha_{ii}^{\left(C\right)}=\frac{1}{\Delta C}\ln\frac{\begin{array}{c} \cos\left(\frac{\pi }{8}\pm (3n\pi r_{m}\alpha_{1}^{\left(C\right)}\Delta C_{1})/2h\right)-\sqrt{\left((2-\sqrt{2})/2\right)}\\ \times \cos\left\{\sin^{-1}\left[\sqrt{2/(2-\sqrt{2})}\sin(\pi /8\pm 3n\pi r_{m}\alpha_{1}^{\left(C\right)}\Delta C_{1}/2h)\right]\right\}+l_{i}/l \end{array}}{(\sqrt{2+\sqrt{2}}-\sqrt{2-\sqrt{2}})/2+l_{i}/l},$$

where the upper and lower signs denote positive and NMEs, respectively. Figure 10 shows—within the context of $E_{1}/E_{2}=\alpha_{1}^{\left(C\right)}/\alpha_{2}^{\left(C\right)}=1$ and Δ*C*_2_ = 0—the effect of change in environmental moisture concentration Δ*C* where *l_i_*/*l* = 1 while layer 2 is water-proofed. Specifically, figure 10*a* shows the effect of various polymer spring CME at Δ*C*_1_ = 0.5Δ*C*, while figure 10*b* exhibits the influence of various polymer absorptivity at $\alpha_{1}^{\left(C\right)}=5\times 10^{-3}$. The range for small deformation model (approx) is confined within Δ*C* ≈ 0 for clarity and also for recognizing that the small deformation model is inaccurate for finite change in the moisture concentration. As expected, the curves for large deformation model (exact) intersects those of the small deformation model at Δ*C* = 0. Perusal to figure 10 suggests that the small deformation model underestimates and overestimates the magnitude of the effective CME under drying and moistening, respectively.

Results plotted in figures 8–10 were calculated based on $\alpha_{1}^{\left(C\right)}>\alpha_{2}^{\left(C\right)}$ and $\alpha_{1}^{\left(T\right)}>\alpha_{2}^{\left(T\right)}$, with the spiral springs attached in the manner furnished in figure 2 so as to give NHTE properties. Suppose either the materials for layers 1 and 2 are swapped or if the ends of the spiral bimaterial spring are attached to different equal-armed crosses—i.e. with reference to figure 2, the inner end of the spiral spring is attached to the ABCD cross while the outer end is attached to the EFGH cross—the metamaterial exhibits positive coefficients of thermal and moisture expansion. In the case of PHTE, the plotted coefficients of thermal and moisture expansions in figures 8–10 would appear as mirror images where the horizontal axes of *α*^(*T*)^ = 0 or *α*^(*C*)^ = 0 are the axes of symmetry.

We have so far considered the original state being *θ*_0_ = 22.5° and *ϕ*_0_ = 45° in complying with the Maltese cross geometry. As one can expect, the overall properties can be adjusted if the angles were to be altered, even if we impose a similar condition 0^°^ < *θ*_0_ < *ϕ*_0_ < 90°. For example, if we let *ϕ*_0_ be large (i.e. close to 90°), a very small change of *dθ* would bring about a much larger *dϕ*, thereby giving a much larger infinitesimal expansion coefficient, but the range of deformation is limited, i.e. the deformation is arrested when *ϕ* reaches 90°.

The length scale depends on the manufacturing technique. It has been reported that aluminium oxide nanoparticles of size 50 nm [56] and more recently iron oxide nanoparticles of size 15 nm [57] have been successfully employed in three-dimensional printing. Suppose a typical nanoparticle size of 30 nm is used for three-dimensional printing, the spiral spring thickness *h*_1_ + *h*_2_ can be of thickness 600 nm, while the pin joint diameter and the rod width can be 3 µm and 6 µm, respectively. If the lengths of the hinge rod and the cross arm be averaged to an order greater than their width, then a choice of *l_h_* = 43.3 µm and *l* = 80 µm would satisfy the first of equation (3.3). If we let *l*_1_ = *l*_2_ = *l*, as was adopted for plotting figures 8*a* and 10, then the size of a repetitive unit is $2X_{1}^{0}=2X_{2}^{0}=247\;\mu m$. In other words, a metamaterial unit of length scale in the order of 10^2^ µm can be achieved by three-dimensional printing using nanoparticle size in the order of 10^1^ nm.

In comparison to a related but simpler metamaterial geometry consisting of counter-rotating crosses without the hinge rods and spiral springs [49], the magnitudes of the current effective CTE and CME are greater by three orders. This is attributed to the spiral bimaterial spring and greater extent of hinge rod rotation in comparison to the cross rotation, which effectively accentuates the hygrothermal strain and, thereby, enhances the coefficients of expansion.

Having plotted the results of effective CTE and CME, it can now be justified why the CTE and CME of the crosses, hinge rods and connecting rods (collectively known as ‘linkages’) can be neglected. With reference to table 1, the CTE of metals is generally in the order of 10^−6^ K^−1^ (to the order of 10^−5^ K^−1^) while perusal to figure 8 indicates that the calculated effective CTE is generally in the order of 10^−3^ K^−1^ (to the order of 10^−2^ K^−1^). Likewise, if we were to select a polymer for the linkages, then its CME would be in the order of 10^−3^ [55] but reference to figure 10 reveals that the calculated effective CME is in the order of 10^0^. In both the CTE and CME cases, if the linkage material is selected from one of the materials of the bimaterial strip, the expansion coefficients are about three orders lower than those of the developed effective expansion coefficients models, thereby justifying the assumption of zero expansion coefficients for the linkages in the analysis.

## 6. Conclusion

A metamaterial inspired by the Maltese cross has been explored herein in which its coefficients of moisture and thermal expansions can be controlled in the following ways:

(a) The determination of positive or negative hygrothermal expansivity can be tailor-made by the choice of bimaterial layer arrangement and its connection to the equal-armed crosses of the metamaterial
(b) The constant CME and CTE for infinitesimal change in environmental condition is influenced by the choice of materials used for the bimaterial spring.
(c) The fine-tuning and imposition of in-plane anisotropy of CME and CTE is achieved by controlling the ratio of *l*_1_/*l* vis-à-vis *l*_2_/*l*.
(d) A greater range for fine-tuning can be achieved by proportionally controlling the number of coils *n* and the mean radius of curvature *r_m_* of the bimaterial spiral spring, as well as by the inversely proportional control of its thickness *h*.

From figures 2 to 3, it can be inferred that in the absence of environmental change, the Maltese cross metamaterial can deform with negative Poisson's ratio characteristics when a force is applied parallel to one of the on-axes directions, with perfect auxeticity *v* = −1 being achieved if the connecting rods aligned along both axes are of the same length. As such, an investigation on the effective Young's modulus and Poisson's ratio of the Maltese cross metamaterial is recommended for future work. As with many other two-dimensional metamaterials that have been extended to three-dimensional, it is herein recommended that the Maltese cross metamaterial be extended to its three-dimensional version for future work.

## Data accessibility

This article does not contain any additional data.
