# Supplementary material for: Adjustable positive and negative hygrothermal expansion metamaterial inspired by the Maltese cross
Source: R Soc Open Sci. 2021 Aug 4;8(8):210593. doi: 10.1098/rsos.210593 (PMC8334837; doi:10.1098/rsos.210593)
Supplement: Supplementary file 1 [file rsos210593_review_history.pdf]

THE ROYAL SOCIETY
PUBLISHING

ROYAL SOCIETY
OPEN SCIENCE

Adjustable positive and negative hygrothermal expansion metamaterial inspired by the Maltese cross

Teik-Cheng Lim

Article citation details
R. Soc. open sci. **8**: 210593.
http://dx.doi.org/10.1098/rsos.210593

Review timeline

Original submission:	8 April 2021
Revised submission:	4 July 2021
Final acceptance:	8 July 2021

Note: Reports are unedited and appear as submitted by the referee. The review history appears in chronological order.

Note: This manuscript was transferred from another Royal Society journal without peer review.

Review History

RSOS-210593.R0 (Original submission)

Review form: Reviewer 1

Is the manuscript scientifically sound in its present form?
Yes

Are the interpretations and conclusions justified by the results?
Yes

Is the language acceptable?
Yes

Do you have any ethical concerns with this paper?
No

Have you any concerns about statistical analyses in this paper?
No

Reports © 2021 The Reviewers; Decision Letters © 2021 The Reviewers and Editors; Responses © 2021 The Reviewers, Editors and Authors. Published by the Royal Society under the terms of the Creative Commons Attribution License http://creativecommons.org/licenses/by/4.0/, which permits unrestricted use, provided the original author and source are credited

Recommendation?
Accept with minor revision (please list in comments)

Comments to the Author(s)
In this manuscript, the author proposed a metamaterial based on the geometry of the Maltese cross. The effective coefficients of moisture and thermal expansions can be engineered in this type of metamaterials, to have either positive or negative values. The analytical mechanics study unveils that finite motion analysis produces more accurate prediction (compared to infinitesimal motion analysis) of the effective coefficients of hygrothermal expansion for large changes in hygrothermal conditions. The methods and results are sound and consistent. The manuscript can be considered for publication in Royal Society Open Science following the author' addressing the following minor issues.

(1) Out of the 32 references in total, 14 reference papers were written by the author himself, and another 5 by Prof J.N. Grima. It may leave the readers an impression that the field of this manuscript is not very active. Please rework the references to be more inclusive and systematic.

(2) Why is the Maltese cross-inspired geometry necessary in the design of hygrothermal metamaterials? Is it better than other simpler geometries which may also induce negative (or positive) effective coefficient of hygrothermal expansion?

(3) Page 8 Line 48: The author mentioned the "conservation of the arc length of the bimaterial strip." Is it valid to omit the axial expansion of the bimaterial strip so that the total arc length stays unchanged? Please comment on the assumptions here and their validity.
How small is the hygrothermal expansion/contraction of the truss members (i.e., the arms and the rods)? Is it valid to omit their contribution to the overall effective coefficient of hygrothermal expansion?

(4) What is the range of length scales for the metamaterial to perform as predicted? Does the author anticipate any available form of experimental validation of this proposed Maltese cross-inspired design?

(5) Can this Maltese cross-inspired layout be extended to 3D space for construction of 3D materials with tunable coefficient of hygrothermal expansion?

(6) Typos and other issues
Page 3 Line 57: "In" should be used in "is such a manner that."
Page 19 Line 41: "bimaterrial"
Figure 4 left: the schematic of spiral does not look perfect.
Figure 6: A′, B′ and K′ are not displayed correctly.

Review form: Reviewer 2

Is the manuscript scientifically sound in its present form?
Yes

Are the interpretations and conclusions justified by the results?
No

Is the language acceptable?
Yes

Do you have any ethical concerns with this paper?
No

Have you any concerns about statistical analyses in this paper?
No

Recommendation?
Accept with minor revision (please list in comments)

Comments to the Author(s)
Date: 21-06-18

The manuscript RSOS-210593
by:
Teik-Cheng Lim
tilted:
Adjustable Positive and Negative Hygrothermal Expansion Metamaterial Inspired by the Maltese Cross.

Info

This manuscript draft presents an analysis of a metamaterial inspired by the Maltese cross. The effective coefficients of moisture and thermal expansion models for this structure were developed for small and large changes in the hygrothermal conditions using approximate and exact motion analyses, respectively.

Recommendation

The manuscript can be recommended for publication in this journal after the Authors consider the minor remarks presented below.

Introduction

The state of art presented by the Authors is medium long (32 references) but there are still lots of interesting papers about metamaterials or structures with negative properties.
Structures or metamaterials should be discussed more precisely in the introduction state of the art review section.
Metamaterials are artificially made materials that do not exist in nature. The term derives from the Greek word meta, meaning beyond. More specifically, metamaterials are composites that have the desired combination of properties that cannot be obtained by combining the properties of their constituents. The term was coined in 1999 by Rodger Walser
The unfortunate fact is that no definition exists that would be universally applicable or universally accepted.

There are a few books about Metamaterials that should be mentioned in the introduction, eg. Maier 2017 and Capolino 2009. Some papers were published in the special issue of journals e.g. Special issue: "Advances in Mechanical Metamaterials" in Materials,
https://www.mdpi.com/journal/materials/special_issues/mechanic_mater

Simple mechanical and thermodynamical models, which show auxetic behavior was found in the 80s of the 20th century (see papers of Gibson; Almgren, Kolpakov, Wojciechowski). Since 1987,

when negative Poisson's ratio foams have been developed by Lakes, it is known that materials and structures showing the negative Poisson's ratio do exist in nature.
The mechanical response of these materials can be drastically changed depending on the number of applied loads, and auxetic materials are expected to have unusual, possibly enhanced geometrical and mechanical characteristics such as synclastic curvature in bending, deformation-dependent permeability, high shear stiffness, indentation resistance, and fracture toughness, and improved damping and sound absorption properties.
Some papers of Gibson and co-authors should be considered as papers important to the manuscript (Gibson 1981, 1982). Lorna Gibson one of the first researchers published works about cellular materials with negative PR. Auxetic material and structures or composite with auxetic phase/layer show better damping properties than materials with positive Poisson's ratio (Strek et al. 2019). Biomaterial metamaterials can exhibit temperature dependency of mechanical properties e.g. thermoauxeticity (Jopek 2018) or anomalous deformation (Strek et al. 2009).

General remark

As it was mentioned by the Author "the Maltese cross metamaterial can deform with negative Poisson's ratio characteristics when a force is applied parallel to one of the on-axis directions". It will be very valuable to paper to present some basic characteristics of PR of structure.

Bimaterial spiral spring complicates the possibility of manufacturing the proposed metamaterial structure. Did Author consider a more simple mechanism than a spiral?

Other minor remarks

P4. L47-49. Is it possible to construct "Maltese cross"-like structures with angles fi and theta which are different from 45 deg and 22.5 deg, respectively? How the properties of these structures will be different?

P17. L3-6. Please add references with common biomaterial strip analyzed in the paper.

P17. T1. Please add to table 1 values of PR and CME for given materials.

P19. L41-44. There are two assumptions: equal Young's modulus and equal CME (see Eq. 5.2), so there is not possible to make general conclusions from results.

Some additional references to consider for extension and improvement of the introduction section

- Maier, Stefan A., World Scientific handbook of metamaterials properties, Volume 2: Elastic, Acoustic, and Seismic Metamaterials Series: World Scientific series in nanoscience and nanotechnology volume 16, World Scientific, 2017
- Filippo Capolino, Theory and Phenomena of Metamaterials (Metamaterials Handbook), 2009.
- Filippo Capolino, Applications of Metamaterials, Series: Metamaterials Handbook, CRC Press, 2009
- Gibson L.J. The Elastic and Plastic Behaviour of Cellular Materials, Dissertation, University of Cambridge, 1981.
- Gibson, L.J.; Ashby, M.F., Schajer, G.S.; Robertson, C.I. The mechanics of two-dimensional cellular materials. Proc. R. Soc. Lond. A 1982, 382, 25-42.
- L. J. Gibson and M. F. Ashby, Materials The Mechanics of Three-Dimensional Cellular, Proc. R. Soc. Lond. A 1982 382, 43-59. doi: 10.1098/rspa.1982.0088

• Strek T., Michalski J., Jopek H., Phys. Status Solidi B, 2019, 256(1), 1800423. DOI:10.1002/pssb.201800423
• T. Strek, P. Kedziora, B. Maruszewski, A. Pozniak, K.V. Tretiakov, K.W. Wojciechowski, Journal of Non-Crystalline Solids, 355, 2009, 1387-1392. DOI: 10.1016/j.jnoncrysol.2008.06.087
• Strek, T., Matuszewska, A., Jopek, H. , Phys. Status Solidi B, 2017, 254(12), 1700103. DOI: 10.1002/pssb.201700103
• Jopek, H., Stręk, T., Materials 2018, 11, 294. DOI: 10.3390/ma11020294

Review form: Reviewer 3

Is the manuscript scientifically sound in its present form?
No

Are the interpretations and conclusions justified by the results?
Yes

Is the language acceptable?
Yes

Do you have any ethical concerns with this paper?
No

Have you any concerns about statistical analyses in this paper?
No

Recommendation?
Major revision is needed (please make suggestions in comments)

Comments to the Author(s)
The manuscript is essentially interesting and shows how one can obtain anomalous behaviour from what is being termed as the 'Maltese Cross' motif as a result of changes in temperature or change in moisture content.

There are a number of aspects in the paper which should be improved. These include:
(1) Assumptions for the model are not clearly stated. For example, the derivation assumes that the ligaments do not change length nor do they bend. The latter assumption is easy to justify if the hinges are friction-less and offer no resistance, otherwise even this assumption needs to be stated, justified, and discussed.

(2) The mathematical model hinges on the validity of the equations 2.3 and 2.4. Are these derived by the author, or quoted from others? If derived by author, can a derivation be presented, and ideally some form of validation?

(3) The main figures in the manuscript, in my opinion, are those in Figure 2 and 3. I would recommend some additional colours to fully distinguish the different component units in the system. This will highlight that the 'cross' is a single unit, but the short ligaments (which are coloured in the same red tone) are not. There are also some errors in the colours in Figure 3 (a grey ligament should be red).

(4) Bimaterial coils seem to be available (see https://en.wikipedia.org/wiki/Bimetallic_strip). Maybe the author could consider experimental verification?

Decision letter (RSOS-210593.R0)

We hope you are keeping well at this difficult and unusual time. We continue to value your support of the journal in these challenging circumstances. If Royal Society Open Science can assist you at all, please don't hesitate to let us know at the email address below.

Dear Professor Lim

On behalf of the Editors, we are pleased to inform you that your Manuscript RSOS-210593 "Adjustable Positive and Negative Hygrothermal Expansion Metamaterial Inspired by the Maltese Cross" has been accepted for publication in Royal Society Open Science subject to minor revision in accordance with the referees' reports. Please find the referees' comments along with any feedback from the Editors below my signature.

We invite you to respond to the comments and revise your manuscript. Below the referees’ and Editors’ comments (where applicable) we provide additional requirements. Final acceptance of your manuscript is dependent on these requirements being met. We provide guidance below to help you prepare your revision.

Please submit your revised manuscript and required files (see below) no later than 7 days from today's (ie 28-Jun-2021) date. Note: the ScholarOne system will ‘lock’ if submission of the revision is attempted 7 or more days after the deadline. If you do not think you will be able to meet this deadline please contact the editorial office immediately.

Please note article processing charges apply to papers accepted for publication in Royal Society Open Science (https://royalsocietypublishing.org/rsos/charges). Charges will also apply to papers transferred to the journal from other Royal Society Publishing journals, as well as papers submitted as part of our collaboration with the Royal Society of Chemistry (https://royalsocietypublishing.org/rsos/chemistry). Fee waivers are available but must be requested when you submit your revision (https://royalsocietypublishing.org/rsos/waivers).

Thank you for submitting your manuscript to Royal Society Open Science and we look forward to receiving your revision. If you have any questions at all, please do not hesitate to get in touch.

Kind regards,
Royal Society Open Science Editorial Office
Royal Society Open Science
openscience@royalsociety.org

on behalf of Dr Adil Al-Mayah (Associate Editor) and R. Kerry Rowe (Subject Editor)
openscience@royalsociety.org

Associate Editor Comments to Author (Dr Adil Al-Mayah):

The reviewers have made some excellent suggestions to improve the quality of the paper and its impact. Cited references need to be modified to include more related work related. Also,

assumptions must be clearly stated. Figures modifications are highly recommended for a clear presentation.

Reviewer comments to Author:
Reviewer: 1
Comments to the Author(s)
In this manuscript, the author proposed a metamaterial based on the geometry of the Maltese cross. The effective coefficients of moisture and thermal expansions can be engineered in this type of metamaterials, to have either positive or negative values. The analytical mechanics study unveils that finite motion analysis produces more accurate prediction (compared to infinitesimal motion analysis) of the effective coefficients of hygrothermal expansion for large changes in hygrothermal conditions. The methods and results are sound and consistent. The manuscript can be considered for publication in Royal Society Open Science following the author' addressing the following minor issues.

(1) Out of the 32 references in total, 14 reference papers were written by the author himself, and another 5 by Prof J.N. Grima. It may leave the readers an impression that the field of this manuscript is not very active. Please rework the references to be more inclusive and systematic.

(2) Why is the Maltese cross-inspired geometry necessary in the design of hygrothermal metamaterials? Is it better than other simpler geometries which may also induce negative (or positive) effective coefficient of hygrothermal expansion?

(3) Page 8 Line 48: The author mentioned the "conservation of the arc length of the bimaterial strip." Is it valid to omit the axial expansion of the bimaterial strip so that the total arc length stays unchanged? Please comment on the assumptions here and their validity.
How small is the hygrothermal expansion/contraction of the truss members (i.e., the arms and the rods)? Is it valid to omit their contribution to the overall effective coefficient of hygrothermal expansion?

(4) What is the range of length scales for the metamaterial to perform as predicted? Does the author anticipate any available form of experimental validation of this proposed Maltese cross-inspired design?

(5) Can this Maltese cross-inspired layout be extended to 3D space for construction of 3D materials with tunable coefficient of hygrothermal expansion?

(6) Typos and other issues
Page 3 Line 57: "In" should be used in "is such a manner that."
Page 19 Line 41: "bimaterrial"
Figure 4 left: the schematic of spiral does not look perfect.
Figure 6: A′, B′ and K′ are not displayed correctly.

Reviewer: 2
Comments to the Author(s)
Date: 21-06-18
The manuscript RSOS-210593
by:
Teik-Cheng Lim
tilted:
Adjustable Positive and Negative Hygrothermal Expansion Metamaterial Inspired by the Maltese Cross.

Info

This manuscript draft presents an analysis of a metamaterial inspired by the Maltese cross. The effective coefficients of moisture and thermal expansion models for this structure were developed for small and large changes in the hygrothermal conditions using approximate and exact motion analyses, respectively.

Recommendation

The manuscript can be recommended for publication in this journal after the Authors consider the minor remarks presented below.

Introduction

The state of art presented by the Authors is medium long (32 references) but there are still lots of interesting papers about metamaterials or structures with negative properties.
Structures or metamaterials should be discussed more precisely in the introduction state of the art review section.
Metamaterials are artificially made materials that do not exist in nature. The term derives from the Greek word meta, meaning beyond. More specifically, metamaterials are composites that have the desired combination of properties that cannot be obtained by combining the properties of their constituents. The term was coined in 1999 by Rodger Walser
The unfortunate fact is that no definition exists that would be universally applicable or universally accepted.

There are a few books about Metamaterials that should be mentioned in the introduction, eg. Maier 2017 and Capolino 2009. Some papers were published in the special issue of journals e.g. Special issue: "Advances in Mechanical Metamaterials" in Materials, https://www.mdpi.com/journal/materials/special_issues/mechanic_mater

Simple mechanical and thermodynamical models, which show auxetic behavior was found in the 80s of the 20th century (see papers of Gibson; Almgren, Kolpakov, Wojciechowski). Since 1987, when negative Poisson's ratio foams have been developed by Lakes, it is known that materials and structures showing the negative Poisson's ratio do exist in nature.
The mechanical response of these materials can be drastically changed depending on the number of applied loads, and auxetic materials are expected to have unusual, possibly enhanced geometrical and mechanical characteristics such as synclastic curvature in bending, deformation-dependent permeability, high shear stiffness, indentation resistance, and fracture toughness, and improved damping and sound absorption properties.
Some papers of Gibson and co-authors should be considered as papers important to the manuscript (Gibson 1981, 1982). Lorna Gibson one of the first researchers published works about cellular materials with negative PR. Auxetic material and structures or composite with auxetic phase/layer show better damping properties than materials with positive Poisson's ratio (Strek et al. 2019). Biomaterial metamaterials can exhibit temperature dependency of mechanical properties e.g. thermoauxeticity (Jopek 2018) or anomalous deformation (Strek et al. 2009).

General remark

As it was mentioned by the Author "the Maltese cross metamaterial can deform with negative Poisson's ratio characteristics when a force is applied parallel to one of the on-axis directions". It will be very valuable to paper to present some basic characteristics of PR of structure.

Bimaterial spiral spring complicates the possibility of manufacturing the proposed metamaterial structure. Did Author consider a more simple mechanism than a spiral?

Other minor remarks

P4. L47-49. Is it possible to construct "Maltese cross"-like structures with angles fi and theta which are different from 45 deg and 22.5 deg, respectively? How the properties of these structures will be different?

P17. L3-6. Please add references with common biomaterial strip analyzed in the paper.

P17. T1. Please add to table 1 values of PR and CME for given materials.

P19. L41-44. There are two assumptions: equal Young's modulus and equal CME (see Eq. 5.2), so there is not possible to make general conclusions from results.

Some additional references to consider for extension and improvement of the introduction section

• Maier, Stefan A., World Scientific handbook of metamaterials properties,
Volume 2: Elastic, Acoustic, and Seismic Metamaterials
Series: World Scientific series in nanoscience and nanotechnology volume 16,
World Scientific, 2017
• Filippo Capolino, Theory and Phenomena of Metamaterials (Metamaterials Handbook), 2009.
• Filippo Capolino, Applications of Metamaterials, Series: Metamaterials Handbook, CRC Press, 2009
• Gibson L.J. The Elastic and Plastic Behaviour of Cellular Materials, Dissertation, University of Cambridge, 1981.
• Gibson, L.J.; Ashby, M.F., Schajer, G.S.; Robertson, C.I. The mechanics of two-dimensional cellular materials. Proc. R. Soc. Lond. A 1982, 382, 25-42.
• L. J. Gibson and M. F. Ashby, Materials The Mechanics of Three-Dimensional Cellular, Proc. R. Soc. Lond. A 1982 382, 43-59. doi: 10.1098/rspa.1982.0088
• Strek T., Michalski J., Jopek H., Phys. Status Solidi B, 2019, 256(1), 1800423. DOI:10.1002/pssb.201800423
• T. Strek, P. Kedziora, B. Maruszewski, A. Pozniak, K.V. Tretiakov, K.W. Wojciechowski, Journal of Non-Crystalline Solids, 355, 2009, 1387-1392. DOI: 10.1016/j.jnoncrysol.2008.06.087
• Strek, T., Matuszewska, A., Jopek, H. , Phys. Status Solidi B, 2017, 254(12), 1700103. DOI: 10.1002/pssb.201700103
• Jopek, H., Stręk, T., Materials 2018, 11, 294. DOI: 10.3390/ma11020294

Reviewer: 3
Comments to the Author(s)

The manuscript is essentially interesting and shows how one can obtain anomalous behaviour from what is being termed as the 'Maltese Cross' motif as a result of changes in temperature or change in moisture content.

There are a number of aspects in the paper which should be improved. These include:

(1) Assumptions for the model are not clearly stated. For example, the derivation assumes that the ligaments do not change length nor do they bend. The latter assumption is easy to justify if the hinges are friction-less and offer no resistance, otherwise even this assumption needs to be stated, justified, and discussed.

(2) The mathematical model hinges on the validity of the equations 2.3 and 2.4. Are these derived by the author, or quoted from others? If derived by author, can a derivation be presented, and ideally some form of validation?

(3) The main figures in the manuscript, in my opinion, are those in Figure 2 and 3. I would recommend some additional colours to fully distinguish the different component units in the system. This will highlight that the 'cross' is a single unit, but the short ligaments (which are coloured in the same red tone) are not. There are also some errors in the colours in Figure 3 (a grey ligament should be red).

(4) Bimaterial coils seem to be available (see https://en.wikipedia.org/wiki/Bimetallic_strip). Maybe the author could consider experimental verification?

===PREPARING YOUR MANUSCRIPT===

Your revised paper should include the changes requested by the referees and Editors of your manuscript. You should provide two versions of this manuscript and both versions must be provided in an editable format:
one version identifying all the changes that have been made (for instance, in coloured highlight, in bold text, or tracked changes);
a 'clean' version of the new manuscript that incorporates the changes made, but does not highlight them. This version will be used for typesetting.
Please ensure that any equations included in the paper are editable text and not embedded images.

Please ensure that you include an acknowledgements' section before your reference list/bibliography. This should acknowledge anyone who assisted with your work, but does not qualify as an author per the guidelines at https://royalsociety.org/journals/ethics-policies/openness/.

While not essential, it will speed up the preparation of your manuscript proof if you format your references/bibliography in Vancouver style (please see https://royalsociety.org/journals/authors/author-guidelines/#formatting). You should include DOIs for as many of the references as possible.

If you have been asked to revise the written English in your submission as a condition of publication, you must do so, and you are expected to provide evidence that you have received language editing support. The journal would prefer that you use a professional language editing service and provide a certificate of editing, but a signed letter from a colleague who is a native speaker of English is acceptable. Note the journal has arranged a number of discounts for authors using professional language editing services (https://royalsociety.org/journals/authors/benefits/language-editing/).

===PREPARING YOUR REVISION IN SCHOLARONE===

To revise your manuscript, log into https://mc.manuscriptcentral.com/rsos and enter your Author Centre - this may be accessed by clicking on "Author" in the dark toolbar at the top of the page (just below the journal name). You will find your manuscript listed under "Manuscripts with Decisions". Under "Actions", click on "Create a Revision".

Attach your point-by-point response to referees and Editors at Step 1 'View and respond to decision letter'. This document should be uploaded in an editable file type (.doc or .docx are preferred). This is essential.

Please ensure that you include a summary of your paper at Step 2 'Type, Title, & Abstract'. This should be no more than 100 words to explain to a non-scientific audience the key findings of your research. This will be included in a weekly highlights email circulated by the Royal Society press office to national UK, international, and scientific news outlets to promote your work.

At Step 3 'File upload' you should include the following files:
-- Your revised manuscript in editable file format (.doc, .docx, or .tex preferred). You should upload two versions:
1) One version identifying all the changes that have been made (for instance, in coloured highlight, in bold text, or tracked changes);
2) A 'clean' version of the new manuscript that incorporates the changes made, but does not highlight them.
-- An individual file of each figure (EPS or print-quality PDF preferred [either format should be produced directly from original creation package], or original software format).
-- An editable file of each table (.doc, .docx, .xls, .xlsx, or .csv).
-- An editable file of all figure and table captions.
Note: you may upload the figure, table, and caption files in a single Zip folder.
-- Any electronic supplementary material (ESM).
-- If you are requesting a discretionary waiver for the article processing charge, the waiver form must be included at this step.
-- If you are providing image files for potential cover images, please upload these at this step, and inform the editorial office you have done so. You must hold the copyright to any image provided.
-- A copy of your point-by-point response to referees and Editors. This will expedite the preparation of your proof.

At Step 6 'Details & comments', you should review and respond to the queries on the electronic submission form. In particular, we would ask that you do the following:
-- Ensure that your data access statement meets the requirements at https://royalsociety.org/journals/authors/author-guidelines/#data. You should ensure that you cite the dataset in your reference list. If you have deposited data etc in the Dryad repository, please only include the 'For publication' link at this stage. You should remove the 'For review' link.
-- If you are requesting an article processing charge waiver, you must select the relevant waiver option (if requesting a discretionary waiver, the form should have been uploaded at Step 3 'File upload' above).
-- If you have uploaded ESM files, please ensure you follow the guidance at https://royalsociety.org/journals/authors/author-guidelines/#supplementary-material to include a suitable title and informative caption. An example of appropriate titling and captioning may be found at https://figshare.com/articles/Table_S2_from_Is_there_a_trade-off_between_peak_performance_and_performance_breadth_across_temperatures_for_aerobic_scope_in_teleost_fishes_/3843624.

At Step 7 'Review & submit', you must view the PDF proof of the manuscript before you will be able to submit the revision. Note: if any parts of the electronic submission form have not been completed, these will be noted by red message boxes.

Author's Response to Decision Letter for (RSOS-210593.R0)

See Appendix A.

Decision letter (RSOS-210593.R1)

We hope you are keeping well at this difficult and unusual time. We continue to value your support of the journal in these challenging circumstances. If Royal Society Open Science can assist you at all, please don't hesitate to let us know at the email address below.

Dear Professor Lim,

I am pleased to inform you that your manuscript entitled "Adjustable Positive and Negative Hygrothermal Expansion Metamaterial Inspired by the Maltese Cross" is now accepted for publication in Royal Society Open Science.

Please ensure that you send to the editorial office an editable version of your accepted manuscript, and individual files for each figure and table included in your manuscript. You can send these in a zip folder if more convenient. Failure to provide these files may delay the processing of your proof. You may disregard this request if you have already provided these files to the editorial office.

If you have not already done so, please remember to make any data sets or code libraries 'live' prior to publication, and update any links as needed when you receive a proof to check - for instance, from a private 'for review' URL to a publicly accessible 'for publication' URL. It is good practice to also add data sets, code and other digital materials to your reference list.

You can expect to receive a proof of your article in the near future. Please contact the editorial office (openscience@royalsociety.org) and the production office (openscience_proofs@royalsociety.org) to let us know if you are likely to be away from e-mail contact -- if you are going to be away, please nominate a co-author (if available) to manage the proofing process, and ensure they are copied into your email to the journal. Due to rapid publication and an extremely tight schedule, if comments are not received, your paper may experience a delay in publication.

Please see the Royal Society Publishing guidance on how you may share your accepted author manuscript at https://royalsociety.org/journals/ethics-policies/media-embargo/. After publication, some additional ways to effectively promote your article can also be found here https://royalsociety.org/blog/2020/07/promoting-your-latest-paper-and-tracking-your-results/.

On behalf of the Editors of Royal Society Open Science, thank you for your support of the journal and we look forward to your continued contributions to Royal Society Open Science.

Kind regards,
Royal Society Open Science Editorial Office
Royal Society Open Science
openscience@royalsociety.org

on behalf of Dr Adil Al-Mayah (Associate Editor) and R. Kerry Rowe (Subject Editor)
openscience@royalsociety.org

Follow Royal Society Publishing on Twitter: @RSocPublishing
Follow Royal Society Publishing on Facebook:
https://www.facebook.com/RoyalSocietyPublishing.FanPage/
Read Royal Society Publishing's blog:
https://royalsociety.org/blog/blogsearchpage/?category=Publishing

Appendix A

List of Amendments on Manuscript ID RSOS-210593

Associate Editor Comments to Author (Dr Adil Al-Mayah):

The reviewers have made some excellent suggestions to improve the quality of the paper and its impact. Cited references need to be modified to include more related work related. Also, assumptions must be clearly stated. Figures modifications are highly recommended for a clear presentation.

Amendment

Thank you for your advice. I have increased the references from 32 to more than 50. Assumptions made have been clearly stated, such as the following in the Concept section.

> $\Delta C > 0$ or temperature increases $\Delta T > 0$, the bimaterial spring becomes more curved. It is assumed that the crosses, hinge rods and connecting rods are rigid so that they do not elongate, shorten, bend or twist, and that the hinges are frictionless so as to permit free rotation at the pin joints. Based on the manner at which the bimaterial spring is attached as

Justification for the assumption appears as the last paragraph of the Results and Discussion section, as shown below.

> Having plotted the results of effective CTE and CME, it can now be justified why the CTE and CME of the crosses, hinge rods and connecting rods (collectively known as "linkages") can be neglected. With reference to table 1, the CTE of metals is generally in the order of $10^{-6}K^{-1}$ (to the order of $10^{-5}K^{-1}$) while perusal to figure 8 indicates that the calculated effective CTE is generally in the order of $10^{-3}K^{-1}$ (to the order of $10^{-2}K^{-1}$). Likewise, if we were to select a polymer for the linkages, then their CME would be in the order of 10^{-3} [55] but reference to figure 10 reveals that the calculated effective CME is in the order of 10^{0}. In both the CTE and CME cases, if the linkage material is selected from one of the materials of the bimaterial strip, the expansion coefficients are about 3 orders lower than those of the developed effective expansion coefficients, thereby justifying the assumption of zero expansion coefficients for the linkages in the analysis.

The figures have been modified.

Reviewer: 1

Comments to the Author(s)

In this manuscript, the author proposed a metamaterial based on the geometry of the Maltese cross. The effective coefficients of moisture and thermal expansions can be engineered in this type of metamaterials, to have either positive or negative values. The analytical mechanics study unveils that finite motion analysis produces more accurate prediction (compared to infinitesimal motion analysis) of the effective coefficients of hygrothermal expansion for large changes in hygrothermal conditions. The methods and results are sound and consistent. The manuscript can be considered for publication in Royal Society Open Science following the author' addressing the following minor issues.

Comment

(1) Out of the 32 references in total, 14 reference papers were written by the author himself, and another 5 by Prof J.N. Grima. It may leave the readers an impression that the field of this manuscript is not very active. Please rework the references to be more inclusive and systematic.

Amendment

The number of references has almost doubled from 32 to 57, notably with the inclusion of works from various pioneers as well as more recent literature, as shown below:

6. Capolino F. 2009 *Theory and phenomena of metamaterials*, Boca Raton: CRC Press. (doi.org/10.1201/9781420054262)
7. Capolino F. 2009 *Applications of metamaterials*, Boca Raton: CRC Press. (doi.org/10.1201/9781420054248)
8. Maier SA. 2017 *World Scientific handbook of metamaterials properties, Volume 2: Elastic, Acoustic, and Seismic Metamaterials Series*, Singapore: World Scientific. (doi.org/10.1142/10642-vol2)
9. Lim TC. 2020 *Mechanics of metamaterials with negative parameters*, Singapore: Springer Nature. (doi:10.1007/978-981-15-6446-8)
10. Kadic M, Frenzel T. 2021 Special Issue "Advances in Mechanical Metamaterials" [Internet]. Basel, Switzerland: MDPI; 2021 [cited 2021 Jul 1]. Available from https://www.mdpi.com/journal/materials/special_issues/mechanic_mater
11. Lim TC 2021. *Special Issue "Metamaterials and Symmetry"* [Internet]. Basel, Switzerland: MDPI; 2021 [cited 2021 Jul 1]. Available from https://www.mdpi.com/journal/symmetry/special_issues/Metamaterials_Symmetry
12. Gibson LJ. 1981 The elastic and plastic behaviour of cellular materials (Doctoral thesis) (doi.org/10.17863/CAM.14049)
13. Gibson LJ, Ashby MF, Schajer GS, Robertson CI. 1982 The mechanics of two-dimensional cellular materials. *Proc. R. Soc. Lond. A* **382**(1782), 25-42 (doi.org/10.1098/rspa.1982.0087)
14. Gibson LJ, Ashby MF. 1982 The nechanics of three-dimensional cellular materials, *Proc. R. Soc. Lond. A* **382**(1782), 43-59. (doi.org/10.1098/rspa.1982.0088)
15. Almgren R. 1985 An isotropic three-dimensional structure with Poisson's ratio =−1. *J. Elast.* **15**(4), 427-430. (doi.org/10.1007/BF00042531)
16. Kolpakov AG. 1985 Determination of the average characteristics of elastic frameworks. *J. Appl. Math. Mech.* **49**(6), 739-745. (doi.org/10.1016/0021-8928(85)90011-5)
17. Wojciechowski KW. 1987 Constant thermodynamic tension Monte Carlo studies of elastic properties of a two-dimensional system of hard cyclic hexamers. *Mol. Phys*. **61**(5), 1247-1258. (doi.org/10.1080/00268978700101761)
18. Wojciechowski KW. 1989 Two-dimensional isotropic system with a negative Poisson ratio. *Phys. Lett. A* **137**(1-2), 60-64. (doi.org/10.1016/0375-9601(89)90971-7)
19. Lakes RS. 1987 Foam structures with a negative Poisson's ratio. *Science* 235(4792), 1038-1040. (doi.org/10.1126/science.235.4792.1038)
20. Strek T, Matuszewska A, Jopek H. 2017 Finite element analysis of the influence of the covering auxetic layer of plate on the contact pressure. *Phys. Status Solidi B* **254**(12), 1700103 (doi.org/10.1002/pssb.201700103)
21. Strek T, Michalski J, Jopek H. 2019 Computational analysis of the mechanical impedance of the sandwich beam with auxetic metal foam core. *Phys. Status Solidi B* **256**(1), 1800423. (doi.org/10.1002/pssb.201800423)
22. Jopek H, Strek T. 2018 Thoemoauxetic behaviour of composite structures. *Materials* **11**(2), 294. (doi.org/10.3390/ma11020294)
23. Strek T, Kedziora P, Maruszewski B, Pozniak A, Tretiakov KV, Wojciechowski KW. 2009 Finite element analysis of auxetic obstacle deformation and fluid flow in a channel. *J. Non-Cryst. Solids* **355**(24-27), 1387-1392. (doi.org/10.1016/j.jnoncrysol.2009.05.032)
24. Strek T, Mariuszewski B, Norojczyk JW, Wojciechowski KW. 2008 Finite element analysis of auxetic plate deformation. J. Non-Cryst. Solids 354(35-39), 4475-4480. (doi.org/10.1016/j.jnoncrysol.2008.06.087)

56. Wu H, Cheng Y, Liu W, He R, Zhou M, Wu S, Song X, Chen Y. 2016 Effect of the particle size and the debinding process on the density of alumina ceramics fabricated by 3D printing based on stereolithography. *Ceram. Int.* **42**(15), 17290-17294. (doi.org/10.1016/j.ceramint.2016.08.024)
57. Domènech B Tan ATL, Jelitto H, Berodt EZ, Blankenburg M, Focke O, Cann J, Tasan CC, Ciacchi LC, Müller M, Furlan KP, Hart AJ, Schneider GA. Strong macroscale supercrystalline structures by 3D printing combined with self-assembly of ceramic functionalized nanoparticles. *Adv. Eng. Mater.* **22**(7), 2000352. (doi.org/10.1002/adem.202000352)

Comment
(2) Why is the Maltese cross-inspired geometry necessary in the design of hygrothermal metamaterials? Is it better than other simpler geometries which may also induce negative (or positive) effective coefficient of hygrothermal expansion?

Amendment
The Maltese cross is not the only way, but its linkage mechanism–as proposed in the paper–permits the rotation of the hinge rods $d\phi$ to a greater extent than the rotation of the cross $d\theta$ as shown in figure 7. This enhances the displacement of the connecting rods, which helps to increase the expansion coefficients. A simpler geometry has been attempted recently [49], but its magnitudes of the CTE and CME are lower by 3 orders in comparison to the current work. This is now added as the second last paragraph of the Results and Discussion section, as furnished below.:

In comparison to a related but simpler metamaterial geometry consisting of counter-rotating crosses without the hinge rods and spiral springs [49], the magnitudes of the current effective CTE and CME are greater by 3 orders. This is attributed to the greater extent of hinge rod rotation in comparison to the cross rotation, which effectively accentuates the hygrothermal strain and, thereby, enhances the coefficients of expansion.

Comment
(3) Page 8 Line 48: The author mentioned the "conservation of the arc length of the bimaterial strip." Is it valid to omit the axial expansion of the bimaterial strip so that the total arc length stays unchanged? Please comment on the assumptions here and their validity. How small is the hygrothermal expansion/contraction of the truss members (i.e., the arms and the rods)? Is it valid to omit their contribution to the overall effective coefficient of hygrothermal expansion?

Amendment
It has previously been shown in the appendix A1 of reference [45] that by using commonly available bimaterial strips, the axial expansion and contraction is negligible, and therefore the omission of axial length change is a valid assumption. For example, if one were to use the copper-steel bimetallic strip and change the temperature by 100K, the magnitude of the percentage error for assuming constant arc length is only 0.138%. This piece of information has been incorporated to the paragraph after equation (2.2) as shown below:

It has previously been shown, in appendix A1 of reference [45], that the change to the arc length is marginal such that the error introduced for assuming constancy in arc length is negligible. For example, in the case of the commonly used copper-steel bimaterial strip, the error for assuming constant arc length is in the order of 10^{-3} [45]. Suppose each equal-armed

The hygrothermal expansion from the crosses, hinge rods and connecting rods can be neglected because their expansion coefficient is 3 orders lower than the calculated expansion coefficients which assumes zero expansion of the cross and the rods. For example, if the crosses and rods were to be selected from any one of the metals listed in table 1 (these are the metals used for the bimaterial strips), then their CTE would be from the order of $10^{-6}K^{-1}$ (to the order of $10^{-5}K^{-1}$). With reference to figure 8, the calculated effective CTE is from the order of $10^{-3}K^{-1}$ (to the order of $10^{-2}K^{-1}$).
Likewise, if we were to select a polymer for the crosses and the rods, then their CME would be in the order of 10^{-3} [55]. Perusal to figure 10 reveals that the calculated effective CME is in the order of 10^{0}. This clarification is now added as the last paragraph of the Results and Discussion section as follows:

Having plotted the results of effective CTE and CME, it can now be justified why the CTE and CME of the crosses, hinge rods and connecting rods (collectively known as "linkages") can be neglected. With reference to table 1, the CTE of metals is generally in the order of $10^{-6}K^{-1}$ (to the order of $10^{-5}K^{-1}$) while perusal to figure 8 indicates that the calculated effective CTE is generally in the order of $10^{-3}K^{-1}$ (to the order of $10^{-2}K^{-1}$). Likewise, if we were to select a polymer for the linkages, then its CME would be in the order of 10^{-3} [55] but reference to figure 10 reveals that the calculated effective CME is in the order of 10^{0}. In both the CTE and CME cases, if the linkage material is selected from one of the materials of the bimaterial strip, the expansion coefficients are about 3 orders lower than those of the developed effective expansion coefficients models, thereby justifying the assumption of zero expansion coefficients for the linkages in the analysis.

Comment
(4) What is the range of length scales for the metamaterial to perform as predicted? Does the author anticipate any available form of experimental validation of this proposed Maltese cross-inspired design?

Amendment
If the metamaterial is built by 3D printing using typical nanoparticle size of order $10^{1}nm$, the size of the metamaterial's repetitive unit can go down to the order of $10^{2}\mu m$. This calculation is now furnished in the 3rd last paragraph of the Results and Discussion section

The length scale depends on the manufacturing technique. It has been reported that aluminum oxide nanoparticles of size $50nm$ [56] and more recently iron oxide nanoparticles of size $15nm$ [57] have been successfully employed in 3D printing. Suppose a typical nanoparticle size of $30nm$ is used for 3D printing, the spiral spring thickness $h_1 + h_2$ can be of thickness $600nm$ while the pin joint diameter and the rod width can be $3\mu m$ and $6\mu m$, respectively. If the lengths of the hinge rod and the cross arm be averaged to an order greater than their width, then a choice of $l_h = 43.3\mu m$ and $l = 80\mu m$ would satisfy the first of equation (3.3). If we let $l_1 = l_2 = l$, as was adopted for plotting figures 8 (left) and figure 10, then the size of a repetitive unit is $2X_1^0 = 2X_2^0 = 247\mu m$. In other words, a metamaterial unit of length scale in the order of $10^2\mu m$ can be achieved by 3D printing using nanoparticle size in the order of $10^1 nm$.

I would anticipate some form of experimental validation in a few years' time.

Comment

(5) Can this Maltese cross-inspired layout be extended to 3D space for construction of 3D materials with tunable coefficient of hygrothermal expansion?

Amendment

It is possible to extend the Maltese cross metamaterial to a 3D version with tunable coefficient of hygrothermal expansion, so I am including this statement as a suggestion for future work in the Conclusion section.

metamaterial is recommended for future work. As with many other 2D metamaterials that have been extended to 3D, it is herein recommended that the Maltese cross metamaterial be extended to its 3D version for future work.

(6) Typos and other issues

Page 3 Line 57: "In" should be used in "is such a manner that."

Page 19 Line 41: "bimaterrial"

Figure 4 left: the schematic of spiral does not look perfect.

Figure 6: A', B' and K' are not displayed correctly.

Amendment

These have been corrected.

Reviewer: 2

Comments to the Author(s)

Date: 21-06-18

The manuscript RSOS-210593
by:
Teik-Cheng Lim
titled:
Adjustable Positive and Negative Hygrothermal Expansion Metamaterial Inspired by the Maltese Cross.

Info

This manuscript draft presents an analysis of a metamaterial inspired by the Maltese cross. The effective coefficients of moisture and thermal expansion models for this structure were developed for small and large changes in the hygrothermal conditions using approximate and exact motion analyses, respectively.

Recommendation

The manuscript can be recommended for publication in this journal after the Authors consider the minor remarks presented below.

Introduction

The state of art presented by the Authors is medium long (32 references) but there are still lots of interesting papers about metamaterials or structures with negative properties. Structures or metamaterials should be discussed more precisely in the introduction state of the art review section.
Metamaterials are artificially made materials that do not exist in nature. The term derives from the Greek word meta, meaning beyond. More specifically, metamaterials are composites that have the desired combination of properties that cannot be obtained by combining the properties of their constituents. The term was coined in 1999 by Rodger Walser
The unfortunate fact is that no definition exists that would be universally applicable or universally accepted.

There are a few books about Metamaterials that should be mentioned in the introduction, eg. Maier 2017 and Capolino 2009. Some papers were published in the special issue of journals e.g. Special issue: "Advances in Mechanical Metamaterials” in Materials, https://www.mdpi.com/journal/materials/special_issues/mechanic_mater

Simple mechanical and thermodynamical models, which show auxetic behavior was found in the 80s of the 20th century (see papers of Gibson; Almgren, Kolpakov, Wojciechowski).

Since 1987, when negative Poisson’s ratio foams have been developed by Lakes, it is known that materials and structures showing the negative Poisson's ratio do exist in nature.
The mechanical response of these materials can be drastically changed depending on the number of applied loads, and auxetic materials are expected to have unusual, possibly enhanced geometrical and mechanical characteristics such as synclastic curvature in bending, deformation-dependent permeability, high shear stiffness, indentation resistance, and fracture toughness, and improved damping and sound absorption properties.
Some papers of Gibson and co-authors should be considered as papers important to the manuscript (Gibson 1981, 1982). Lorna Gibson one of the first researchers published works about cellular materials with negative PR. Auxetic material and structures or composite with auxetic phase/layer show better damping properties than materials with positive Poisson’s ratio (Strek et al. 2019). Biomaterial metamaterials can exhibit temperature dependency of mechanical properties e.g. thermoauxeticity (Jopek 2018) or anomalous deformation (Strek et al. 2009).

Amendment
The author thanks the reviewer for suggesting a good historical background on metamaterials and auxetic systems, as well as the more recent literature. The Introduction section has been expanded as shown below:

Metamaterials are materials whose micro-lattices are tailor-made so that effective characteristics are predominantly regulated by their microstructural lay-out instead of those by the base materials. Metamaterials are artificially-made materials that do not exist in nature. The term derives from the Greek word meta, meaning beyond. More specifically, metamaterials are composites that have the desired combination of properties that cannot be obtained by combining the properties of their constituents. The term was coined by Rodger Walser. The reader is referred to books that deal with electromagnetic metamaterials and their applications [6,7], elastic, acoustic and seismic metamaterials [8], and negative mechanical metamaterials [9], as well as special issues pertaining to metamaterials [10,11], to name a few. Gibson [12], Gibson *et al*. [13] and Gibson and Ashby [14] are one of the earliest to publish work on cellular materials with auxetic (negative Poisson’s ratio) behaviour. Other pioneering works include those by Almgren [15], Kolpakov [16] and Wojciechowski [17,18], based on mechanical and thermodynamical models. Since 1987, when negative Poisson’s ratio foams have been developed by Lakes [19], it is known that materials and structures showing the negative Poisson's ratio do exist in nature. The mechanical response of these materials can be drastically changed depending on the number of applied loads, and auxetic materials are expected to have unusual, possibly enhanced geometrical and mechanical characteristics such as synclastic curvature in bending, deformation-dependent permeability, high shear stiffness, indentation resistance, and fracture toughness, and improved damping and sound absorption properties. Strek *et al*. [20] studied the contact problem of a composite plate covered with an auxetic layer. Auxetic material and structures or composite with auxetic phase/layer show better damping properties than materials with positive Poisson’s ratio [21]. Some metamaterials have been shown to exhibit temperature dependency of mechanical properties, such as thermoauxeticity [22] or anomalous deformation [23,24]. Owing to the

General remark

As it was mentioned by the Author “the Maltese cross metamaterial can deform with negative Poisson’s ratio characteristics when a force is applied parallel to one of the on-axis directions”. It will be very valuable to paper to present some basic characteristics of PR of structure.

Amendment

The author thanks the reviewer for suggesting to explore the auxetic properties. A paper on mechanical properties of this metamaterial has just been submitted to a journal for consideration. As such, a brief remark is made in the Conclusion section as follows:

> From figures 2 or 3, it can be inferred that in the absence of environmental change, the Maltese cross metamaterial can deform with negative Poisson’s ratio characteristics when a force is applied parallel to one of the on-axes directions, with perfect auxeticity $v = -1$ being achieved if the connecting rods aligned along both axes are of the same length. As such, an investigation on the effective Young’s modulus and Poisson’s ratio of the Maltese cross metamaterial is recommended for future work.

Comment

Bimaterial spiral spring complicates the possibility of manufacturing the proposed metamaterial structure. Did Author consider a more simple mechanism than a spiral?

Amendment

Yes, the author has considered a simpler mechanism consisting of a pair of counter-rotating crosses without the spiral spring, and the work was recently reported in reference [49], but the magnitudes of its CTE and CME are lower by 3 orders in comparison to those developed in this manuscript. This is now updated as the second last paragraph of the Results and Discussion section, as furnished below:

> In comparison to a related but simpler metamaterial geometry consisting of counter-rotating crosses without the hinge rods and spiral springs [49], the magnitudes of the current effective CTE and CME are greater by 3 orders. This is attributed to the greater extent of hinge rod rotation in comparison to the cross rotation, which effectively accentuates the hygrothermal strain and, thereby, enhances the coefficients of expansion.

Other minor remarks

Comment

P4. L47-49. Is it possible to construct “Maltese cross”-like structures with angles fi and theta which are different from 45 deg and 22.5 deg, respectively? How the properties of these structures will be different?

Amendment

Yes, it is possible to construct "Maltese cross"-like structures with angles fi and theta which are different from 45 deg and 22.5 deg, respectively. This is now briefly discussed in the 4th last paragraph of the Result and Discussion section:

> We have so far considered the original state being $\theta_0 = 22.5°$ and $\phi_0 = 45°$ in complying to the Maltese cross geometry. As one can expect, the overall properties can be adjusted if the angles were to be altered, even if we impose a similar condition $0° < \theta_0 < \phi_0 < 90°$. For example, if we let ϕ_0 be large (i.e. close to 90°), a very small change of $d\theta$ would bring about a much larger $d\phi$, thereby giving a much large infinitesimal expansion coefficient, but the range of deformation is limited, i.e. the deformation is arrested when ϕ reaches 90°.

Comment

P17. L3-6. Please add references with common bimaterial strip analyzed in the paper.

Amendment

The common bimaterial strip analyzed in the paper are corresponded to references [51-54]. The following has been amended just before table 1.

> (4.4) and (4.6). The most common bimaterial strip for responding to temperature change is made from copper–steel (C-S) pair [51-54]. To evaluate the effect of CTE difference, other material pairs such as tungsten–silicon carbide (T-SC) pair and brass–titanium (B-T) pair are included [3,45]. The relevant properties for these materials are furnished in table 1, in which

51. Katrus OA, Aleshina AV, Gribkov VK, Ocheretyanskii VM. 1984 Some properties of thin steel-copper bimetal sheet. *Soviet Powder Metall. Metal Ceram*. **23**(5), 370-372. (doi.org/10.1007/BF00796600)
52. Tan C, Zhou K, Ma W, Min L. 2018 Interfacial characteristic and mechanical performance of maraging steel-copper functional bimetal produced by selective laser melting based hybrid manufacture. *Mater. Des*. **155**, 77-85. (doi.org/10.1016/j.matdes.2018.05.064)
53. Osipovich KS, Chumaevskii AV, Eliseev AA, Kalashnikov KN, Kolubaev EA, Rubtsov VE, Astafurova EG. 2019 Peculiarities of structure formation in copper/steel bimetal fabricated by electron-beam additive technology. *Russian Phys. J*. **62**(8), 1486-1494. (doi.org/10.1007/s11182-019-01867-w)
54. Wang Y, Gao Y, Li Y, Zhai W, Sun L, Zhang C. 2019 Review of preparation and application of copper–steel bimetal composites. *Emerging Mater. Res*. **8**(4), 538-551. (doi.org/10.1680/jemmr.17.00008)

Comment

P17. T1. Please add to table 1 values of PR and CME for given materials.

Amendment

The PR of the metals have been added. However, their CME is undefined because the metals do not absorb moisture.

Table 1. Material properties for the tungsten–silicon carbide (T-SC), copper–steel (C-S) and brass–titanium (B-T) bimaterial strips.

Bimaterial pair	CTE	Young's modulus	Poisson's ratio
Tungsten<br>Silicon Carbide	$\alpha_1^{(T)} = 4.5 \times 10^{-6} K^{-1}$<br>$\alpha_2^{(T)} = 2.77 \times 10^{-6} K^{-1}$	$E_1 = 405\ GPa$<br>$E_2 = 450\ GPa$	$v_1 = 0.280$<br>$v_2 = 0.360$
Copper<br>Steel	$\alpha_1^{(T)} = 17 \times 10^{-6} K^{-1}$<br>$\alpha_2^{(T)} = 12 \times 10^{-6} K^{-1}$	$E_1 = 117\ GPa$<br>$E_2 = 200\ GPa$	$v_1 = 0.360$<br>$v_2 = 0.300$
Brass<br>Titanium	$\alpha_1^{(T)} = 19 \times 10^{-6} K^{-1}$<br>$\alpha_2^{(T)} = 8.6 \times 10^{-6} K^{-1}$	$E_1 = 112.5\ GPa$<br>$E_2 = 110.3\ GPa$	$v_1 = 0.357$<br>$v_2 = 0.300$

Comment

P19. L41-44. There are two assumptions: equal Young's modulus and equal CME (see Eq. 5.2), so there is not possible to make general conclusions from results.

Amendment

The subsequent discussion, after equation (5.4), has been rephrased to confine the validity within the context of $E_1/E_2 = \alpha_1^{(C)}/\alpha_2^{(C)} = 1$ and $\Delta C_2 = 0$, as shown below.

respectively. Figure 10 shows that–within the context of $E_1/E_2 = \alpha_1^{(C)}/\alpha_2^{(C)} = 1$ and $\Delta C_2 = 0$–the effect of

Comment

Some additional references to consider for extension and improvement of the introduction section

• Maier, Stefan A., World Scientific handbook of metamaterials properties,
Volume 2: Elastic, Acoustic, and Seismic Metamaterials
Series: World Scientific series in nanoscience and nanotechnology volume 16,
World Scientific, 2017

• Filippo Capolino, Theory and Phenomena of Metamaterials (Metamaterials Handbook), 2009.

• Filippo Capolino, Applications of Metamaterials, Series: Metamaterials Handbook, CRC Press, 2009

• Gibson L.J. The Elastic and Plastic Behaviour of Cellular Materials, Dissertation, University of Cambridge, 1981.

• Gibson, L.J.; Ashby, M.F., Schajer, G.S.; Robertson, C.I. The mechanics of two-dimensional cellular materials. Proc. R. Soc. Lond. A 1982, 382, 25-42.

• L. J. Gibson and M. F. Ashby, Materials The Mechanics of Three-Dimensional Cellular, Proc. R. Soc. Lond. A 1982 382, 43-59. doi: 10.1098/rspa.1982.0088

• Strek T., Michalski J., Jopek H., Phys. Status Solidi B, 2019, 256(1), 1800423. DOI:10.1002/pssb.201800423

• T. Strek, P. Kedziora, B. Maruszewski, A. Pozniak, K.V. Tretiakov, K.W. Wojciechowski, Journal of Non-Crystalline Solids, 355, 2009, 1387-1392. DOI: 10.1016/j.jnoncrysol.2008.06.087

• Strek, T., Matuszewska, A., Jopek, H. , Phys. Status Solidi B, 2017, 254(12), 1700103. DOI: 10.1002/pssb.201700103
• Jopek, H., Stręk, T., Materials 2018, 11, 294. DOI: 10.3390/ma11020294

Amendment

The above literature (plus a few more) have been added, and can be found in References [6-24].

Reviewer: 3

Comments to the Author(s)

The manuscript is essentially interesting and shows how one can obtain anomalous behaviour from what is being termed as the 'Maltese Cross' motif as a result of changes in temperature or change in moisture content.

There are a number of aspects in the paper which should be improved. These include:

Comment

(1) Assumptions for the model are not clearly stated. For example, the derivation assumes that the ligaments do not change length nor do they bend. The latter assumption is easy to justify if the hinges are friction-less and offer no resistance, otherwise even this assumption needs to be stated, justified, and discussed.

Amendment

The assumptions have been explicitly incorporated into the first paragraph of the Concept section as shown below:

$\Delta C > 0$ or temperature increases $\Delta T > 0$, the bimaterial spring becomes more curved. It is assumed that the crosses, hinge rods and connecting rods are rigid so that they do not elongate, shorten, bend or twist, and that the hinges are frictionless so as to permit free rotation at the pin joints. Based on the manner at which the bimaterial spring is attached as

Comment

(2) The mathematical model hinges on the validity of the equations 2.3 and 2.4. Are these derived by the author, or quoted from others? If derived by author, can a derivation be presented, and ideally some form of validation?

Explanation

The equations (2.3) and (2.4) are inferred from Timoshenko's (1925) work. Upon changing the symbols used, the 2nd equation from the top of page 235 of Timoshenko (1925) reads

$$\frac{1}{r_m} = \frac{\left(\alpha_1^{(T)} - \alpha_2^{(T)}\right)\Delta T}{\frac{h}{2} + \frac{2}{h}(E_1 I_1 + E_2 I_2)\left(\frac{1}{E_1 h_1} + \frac{1}{E_2 h_2}\right)}$$

This is for the case where the bimaterial strip is initially straight and thereafter bent to a curvature of $1/r_m$ upon temperature change of ΔT. In other words, the curvature changes from zero to $1/r_m$, and one may write this change of curvature as

$$\frac{1}{r_m} - \frac{1}{\infty} = \frac{\left(\alpha_1^{(T)} - \alpha_2^{(T)}\right)\Delta T}{\frac{h}{2} + \frac{2}{h}(E_1 I_1 + E_2 I_2)\left(\frac{1}{E_1 h_1} + \frac{1}{E_2 h_2}\right)}$$

where $1/\infty$ is the initially zero curvature. Timoshenko then went on to simplify the above by considering $h_1 = h_2$ and $E_1 = E_2$ to give

$$\frac{1}{r_m} = \frac{3}{2}\frac{\left(\alpha_1^{(T)} - \alpha_2^{(T)}\right)\Delta T}{h} \tag{6}$$

which appears as equation (6) of his paper. See snapshot of Timoshenko's (1925) paper on page 235.

Sept., 1925] BI-METAL THERMOSTATS 235

using (1) and (3)

$$\frac{h}{2\rho}+\frac{2(E_1I_1+E_2I_2)}{h\rho}\left(\frac{1}{E_1a_1}+\frac{1}{E_2a_2}\right)=(\alpha_2-\alpha_1)\,(t-t_0)$$

From which,

$$\frac{1}{\rho}=\frac{(\alpha_2-\alpha_1)\,(t-t_0)}{\frac{h}{2}+\frac{2(E_1I_1+E_2I_2)}{h}\left(\frac{1}{E_1a_1}+\frac{1}{E_2a_2}\right)}$$

Letting,

$$\frac{a_1}{a_2}=m\ ,\quad \frac{E_1}{E_2}=n\ ,$$

and remembering that

$$I_1=\frac{a_1^3}{12}\ ,\qquad I_2=\frac{a_2^3}{12}$$

the following general equation for the curvature of a bi-metal strip will be obtained,

$$\frac{1}{\rho}=\frac{6(\alpha_2-\alpha_1)\,(t-t_0)\,(1+m)^2}{h\left(3(1+m)^2+(1+mn)\left(m^2+\frac{1}{mn}\right)\right)} \tag{4}$$

If the thicknesses of both metals are equal,

$$a_1=a_2\ ,\quad m=1$$

$$\frac{1}{\rho}=\frac{24(\alpha_2-\alpha_1)\,(t-t_0)}{h(14+n+1/n)} \tag{5}$$

The curvature is proportional to the difference in elongation of the two metals and inversely proportional to the thickness of the strip. It is seen that the magnitude of the ratio $\frac{E_1}{E_2}=n$ does not produce any substantial effect on the curvature of the strip. Take for instance, $n=1$, then,

$$\frac{1}{\rho}=\frac{3}{2}\frac{(\alpha_2-\alpha_1)\,(t-t_0)}{h} \tag{6}$$

Many of the subsequent example cases (except for buckling problems) adopted equation (6), presumably for the sake of brevity. Refer now to figure 9 with caption "Bending of curved bi-metal strip" on page 242, in which Timoshenko considered a bi-metallic strip with the initial

curvature $1/r_m$ (indicated as $1/\rho_0$) with the material with larger coefficient of expansion on the concave side. See screenshot below for page 242.

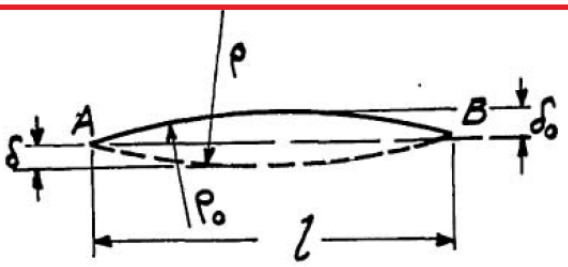

FIG. 9. *Bending of curved bi-metal strip.*

It is clear that the bending of the plate into a spherical form is impossible without some strain in the middle surface of the plate. If the deflections are small, say less than a half of the thickness of the plate, this strain in the middle surface of the plate can be neglected without any essential error, but with the increasing of the deflection, the effect of this strain becomes more and more pronounced and must be taken into consideration.

B. ANALYSIS OF THE STRIP TYPE OF BI-METAL THERMOSTAT

5. *Free bending of bi-metal strip.* Let a strip of bi-metal have an initial curvature equal to $1/\rho_0$ an initial deflection δ_0 (Fig. 9) and be bent in such a manner that the material with the larger coefficient of

During heating the bi-metallic strip becomes less curve and eventually goes over to the other side to form a new curvature of $1/r'_m$ (indicated as $1/\rho$) on figure 9 of Timoshenko (1925). So the total change in curvature becomes

$$\frac{1}{r_m} + \frac{1}{r'_m} = \frac{3}{2}\frac{\left(\alpha_1^{(T)} - \alpha_2^{(T)}\right)\Delta T}{h} \qquad (15)$$

with reference to his equation (6). See screenshot below for page 243.

Sept., 1925] BI-METAL THERMOSTATS 243

expansion is on the concave side. During heating of this strip the curvature will gradually diminish and at a certain temperature the strip will pass through the plane form and afterwards become curved in a downward direction as shown by the dotted line in Fig. 9. The variation of the curvature will be obtained from equation (6)

$$\frac{1}{\rho_0} + \frac{1}{\rho} = \frac{3}{2}\frac{(\alpha_2 - \alpha_1)(t - t_0)}{h} \qquad (15)$$

Since the new curvature takes place on the opposite side from the initial curvature, the change in curvature was obtained by adding up of the magnitudes of the initial curvature and the new curvature, as indicated in equation (15) of Timoshenko's (1925) work.

In the author's current paper, the material with larger coefficient of expansion is placed at the convex side, such that during heating the curvature increases from $1/r_m$ to $1/r'_m$, as illustrated below.

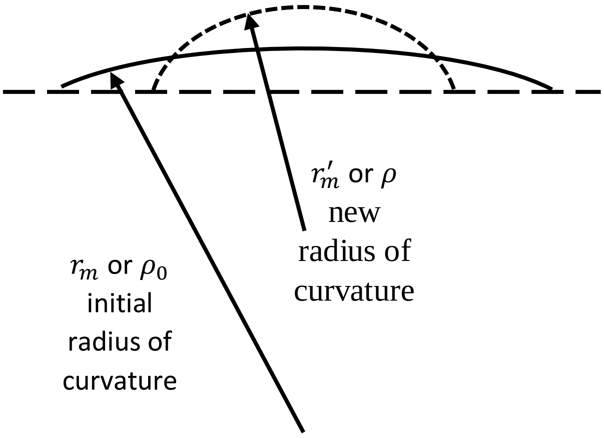

The change in curvature is thus

$$\frac{1}{r'_m} - \frac{1}{r_m} = \frac{3}{2}\frac{\left(\alpha_1^{(T)} - \alpha_2^{(T)}\right)\Delta T}{h}$$

to give a positive value on the LHS. (*Under cooling, i.e.* ΔT *being negative, the curvature decreases to give a correspondingly negative value on the LHS*). Suppose we do not assume $h_1 = h_2$ and $E_1 = E_2$, then the RHS of the above equation must revert to the RHS of the original expression such that equation (2.4) of the current author's work is recovered. If an environmental moisture concentration change of ΔC brings about moisture concentration changes of ΔC_1 and ΔC_2 on the convex and concave layers, respectively, then equation (2.3) is obtained.

Amendment

The sentence before equation (2.3) has been rephrased to more clearly reflect that equations (2.3) and (2.4) are inferred from Timoshenko's (1925) work, particularly from the section on initially curved bi-metallic strips, as shown below:

> respectively. Consider a bimaterial spiral spring of CME $\alpha_1^{(C)}$ and $\alpha_2^{(C)}$, CTE $\alpha_1^{(T)}$ and $\alpha_2^{(T)}$, Young's modulus E_1 and E_2, and thickness h_1 and h_2 corresponding to layers 1 and 2, respectively, the changes in its mean curvature arising from changes in the environmental moisture concentration ΔC and temperature ΔT are inferred from Timoshenko's [50] derivation for an initially curved bimetallic strip to give

Comment

(3) The main figures in the manuscript, in my opinion, are those in Figure 2 and 3. I would recommend some additional colours to fully distinguish the different component units in the system. This will highlight that the 'cross' is a single unit, but the short ligaments (which are coloured in the same red tone) are not. There are also some errors in the colours in Figure 3 (a grey ligament should be red).

Amendment

The short ligaments have been recoloured, and the originally incorrect colour has been corrected.

Comment
(4) Bimaterial coils seem to be available (see https://en.wikipedia.org/wiki/Bimetallic_strip). Maybe the author could consider experimental verification?

Response
Thank you for referring me to this Wikipedia page. I have been given only 7 days make amendments, and have been confined to working from home (due to Covid-19) with no foreseeable date for returning to campus. As such, it is not possible to provide experimental verification within this time frame, other than making inference from Timoshenko's work on initially curved bimetallic strip.
